# Insight into the Nature of the ZnO_*x*_ Promoter during Methanol Synthesis

**DOI:** 10.1021/acscatal.1c05101

**Published:** 2022-05-20

**Authors:** Remco Dalebout, Laura Barberis, Giorgio Totarella, Savannah J. Turner, Camille La Fontaine, Frank M. F. de Groot, Xavier Carrier, Ad M. J. van der Eerden, Florian Meirer, Petra E. de Jongh

**Affiliations:** † Materials Chemistry and Catalysis, Debye Institute for Nanomaterials Science, Utrecht University, Universiteitsweg 99, 3584 CG Utrecht, The Netherlands; ‡ Synchrotron SOLEIL, L’Orme des Merisiers, Saint-Aubin BP 48, Gif-sur-Yvette 91192 CEDEX, France; § Laboratoire de Réactivité de Surface, UMR CNRS 7197, Sorbonne Université, 4 place Jussieu, Paris 75252 CEDEX 05, France; ∥ Inorganic Chemistry and Catalysis, Debye Institute for Nanomaterials Science, Utrecht University, Universiteitsweg 99, 3584 CG Utrecht, The Netherlands

**Keywords:** methanol synthesis, CO hydrogenation, CO_2_, zinc oxide promotion, carbon support, silica, X-ray absorption spectroscopy, copper nanoparticles

## Abstract

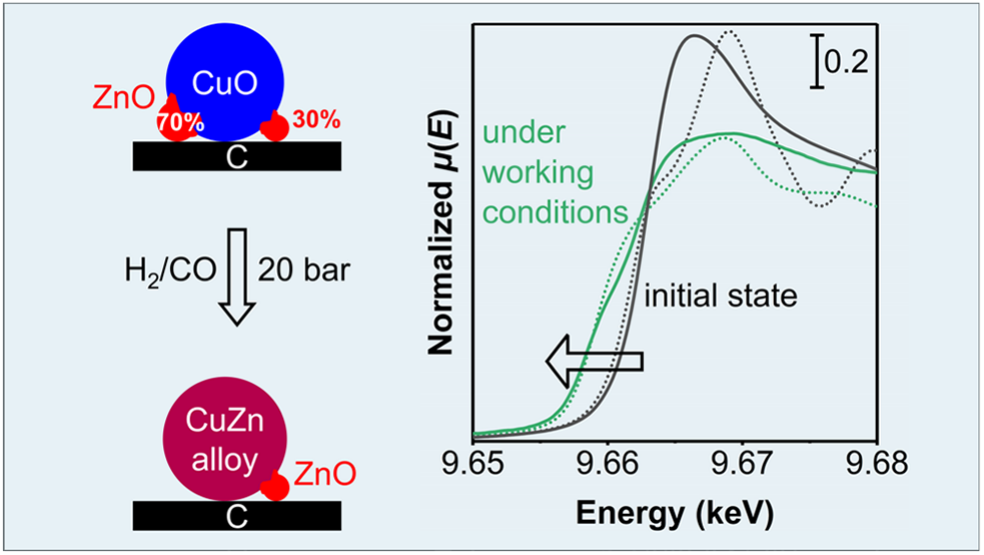

Despite the great commercial relevance of zinc-promoted copper catalysts for methanol synthesis, the nature of the Cu–ZnO_*x*_ synergy and the nature of the active Zn-based promoter species under industrially relevant conditions are still a topic of vivid debate. Detailed characterization of the chemical speciation of any promoter under high-pressure working conditions is challenging but specifically hampered by the large fraction of Zn spectator species bound to the oxidic catalyst support. We present the use of weakly interacting graphitic carbon supports as a tool to study the active speciation of the Zn promoter phase that is in close contact with the Cu nanoparticles using time-resolved X-ray absorption spectroscopy under working conditions. Without an oxidic support, much fewer Zn species need to be added for maximum catalyst activity. A 5–15 min exposure to 1 bar H_2_ at 543 K only slightly reduces the Zn(II), but exposure for several hours to 20 bar H_2_/CO and/or H_2_/CO/CO_2_ leads to an average Zn oxidation number of +(0.5–0.6), only slightly increasing to +0.8 in a 20 bar H_2_/CO_2_ feed. This means that most of the added Zn is in a zerovalent oxidation state during methanol synthesis conditions. The Zn average coordination number is 8, showing that this phase is not at the surface but surrounded by other metal atoms (whether Zn or Cu), and indicating that the Zn diffuses into the Cu nanoparticles under reaction conditions. The time scale of this process corresponds to that of the generally observed activation period for these catalysts. These results reveal the speciation of the relevant Zn promoter species under methanol synthesis conditions and, more generally, present the use of weakly interacting graphitic supports as an important strategy to avoid excessive spectator species, thereby allowing us to study the nature of relevant promoter species.

## 1 Introduction

Methanol synthesis is an important, decades-old industrial process. Nowadays, a coprecipitated Cu/ZnO/Al_2_O_3_ catalyst is used to hydrogenate CO_2_ to methanol in a CO-rich environment. It has been well established that the methanol is predominantly formed from CO_2_ rather than from CO. The role of the CO is to supply CO_2_*via* the reaction with water, which also keeps the water level low.^1−6^ Generally accepted is that Cu is the main active component where ZnO_*x*_ plays a crucial role in promoting the catalyst activity with about an order of magnitude.^5,7−11^ Yet, the exact role of the ZnO_*x*_ promoter is still under debate,^12−14^ especially due to a lack of detailed knowledge on the ZnO_*x*_ speciation, structure, and its interaction with Cu under the typical methanol synthesis conditions at 473–573 K and 20–100 bar.^15,16^

Various hypotheses exist to explain the role of the ZnO_*x*_ promotion. It has been suggested that ZnO_*x*_ increases the Cu dispersion and thereby the active Cu surface area^17,18^ and that the promoter supplies hydrogen to the Cu surface by spillover.^19,20^ The oxidation state of ZnO_*x*_ can also play a role in the morphological change of small Cu particles due to a varying degree of the Cu–ZnO_*x*_ interaction, thereby varying the exposed Cu surface planes.^21^ However, by now it is broadly accepted that the coverage of Cu nanoparticles with partially reduced ZnO is essential for the enhanced methanol production. An open question is still whether the promotion is due to the formation of a ZnO_*x*_ layer on the Cu particles,^19,22−24^ to the formation and migration of Zn atoms on (or into) the Cu surface,^10,13,25,26^ or to the creation of active defects upon Cu–ZnO_*x*_ interaction.^19,23,27^ Research is typically performed on catalysts supported on metal oxides, which may obscure the active ZnO_*x*_ phase by the formation of mixed Zn metal oxides and hence may significantly differ from the relevant speciation and distribution of the active fraction of the ZnO_*x*_ promoter.

It is generally accepted that ZnO_*x*_ (partially) covers the Cu nanoparticles in reducing conditions. The fractional coverage of Cu with ZnO_*x*_ during reaction conditions is mainly influenced by three factors: the feed composition, governing the degree of ZnO_*x*_ reduction; the ZnO_*x*_ loading; and the Cu particle size. For example, Kuld et al.^13^ showed that by applying various feeds during catalyst activation an optimal Zn coverage over a Cu surface of 0.47 was achieved using a Cu/ZnO/Al_2_O_3_ catalyst of constant composition during CO/CO_2_ hydrogenation at ambient pressure. Yet, contradictory results for the optimal Zn coverage were reported by varying the ZnO_*x*_ loading under different reaction conditions.^28,29^ Also in a pure H_2_/CO_2_ feed, an optimal Zn coverage of 0.20, or an atomic Zn/Cu ratio of 1.2–1.6, was reported for Cu/ZnO catalysts.^19,22,23,30,31^ The question remains what the actual state of the ZnO_*x*_ is during working conditions in different feeds at high pressure (e.g., H_2_/CO feed), syngas enriched with a relevant amount of CO_2_ (2–6 vol %^1−5^), or an H_2_/CO_2_ feed.

Much effort has been devoted to studying the interaction and oxidation state of ZnO_*x*_ species in CuZn-based catalysts in the calcined state^29,32−34^ and before/after^33,35−39^ or during^14,32,40−43^ exposure to reducing atmospheres at (near-)ambient pressures (up to 8 bar). On the basis of those results, it is still inconclusive whether the oxidation state of ZnO_*x*_ slightly changes^35,36,40^ and whether Cu–Zn alloys are formed^14,38,44^ or not.^33,35,41,42^ For example, recent studies reported the formation of a Cu–Zn alloy in a Cu/ZnO/Al_2_O_3_ catalyst during a (CO_2_/)H_2_ treatment at 15 bar and 533 K,^45^ but this alloy formation was absent for a Cu/ZnO/faujasite catalyst with almost a 1-to-1 ratio of Cu and Zn.^46^ A recently developed, unique tool that allows us to gain insight into the Zn oxidation state and speciation under realistic high-pressure conditions and in the working state is X-ray absorption spectroscopy (XAS). Very recently, Divins et al.^24^ published an interesting *operando* study at 20–40 bar in a CO_2_-enriched syngas feed using silica and alumina supports, ascribing the active ZnO_*x*_ speciation to a distorted ZnO_*x*_ phase with a maximum content of 9 at% Zn^0^ atoms but most of the Zn species present as metal oxides.

A major obstacle to study the nature of the active site of the ZnO_*x*_ promoter is the strong interaction of the promoter with the oxidic catalyst support, which leads to the formation of a large fraction of Zn spectator species present as formates, oxides, or mixed metal phases.^24,37,45−49^ Hence, the active promoter species represents only a fraction of the Zn species present in the system, and averaged information, such as the Zn oxidation state and coordination number, are not representative for the active ZnO_*x*_ promoter species.

We present graphitic carbon as a support with very limited interaction with Cu and ZnO_*x*_.^47,48^ In combination with a relatively low ZnO_*x*_ loading, it allows us to study specifically the ZnO_*x*_ in contact with the Cu nanoparticles during methanol synthesis and its speciation and interaction with the Cu, based on time-resolved XAS experiments under working conditions, also as a function of different feed compositions.

## 2 Experimental Section

### 2.1 Catalyst Synthesis

A series of CuZnO_*x*_/C catalysts, with similar Cu weight loadings (8.0 ± 0.4 wt %) but varying Zn/Cu molar ratios, were prepared *via* incipient wetness impregnation following a published method.^5^ In brief, powdered high-surface-area graphite (TIMREX E-HSAG500, TIMCAL Graphite & Carbon) was dried at *ca.* 443 K under dynamic vacuum for 1.5 h. The support was impregnated at room temperature under static vacuum to 95% of the total pore volume with an acidified aqueous solution containing 1.8 M copper nitrate (Acros Organics, 99%) and 0–1.8 M zinc nitrate (Sigma-Aldrich, ≥99%). Subsequently, the impregnated support was dried overnight at room temperature under dynamic vacuum and further reduced at 503 K (ramp 2 K min^–1^) in a 100 mL min^–1^ flow of 20 vol % H_2_/N_2_ for 2.5 h. After cooling to room temperature, the sample was exposed to a flow of 100 mL min^–1^ flow of 5 vol % O_2_/N_2_ for 1 h, heated to 473 K with a ramp of 1 K min^–1^ and oxidized at 473 K in 15 vol % O_2_/N_2_ for 1 h.

The Cu/C (8.1 wt % Cu), ZnO_*x*_/C (9.9 wt % ZnO), and CuZnO_*x*_/SiO_2_ catalysts were synthesized following the same procedure as for the CuZnO_*x*_/C catalysts using the respective metal nitrate(s). A different heat treatment was applied only for the ZnO_*x*_/SiO_2_ catalyst (10.0 wt % ZnO): the dried impregnate was heated to 723 K (ramp 2 K min^–1^) in a 200 mL min^–1^ g_cat_^–1^ flow of 2 vol % NO/inert for 1 h.^50^ Both SiO_2_-based catalysts were supported on silica gel (25–75 μm, Davisil, grade 643, Sigma-Aldrich, ≥99%). All catalysts are named CuZn-*X*/C or CuZn-*X*/SiO_2_, in which *X* represents the molar Zn/(Cu + Zn) ratio expressed as percentage and is based on the nominal loading. A commercial Cu/ZnO/Al_2_O_3_/MgO catalyst from Alfa Aesar, containing a Cu/Zn/Al/Mg ratio of 63.8/24.8/10.1/1.3 wt %, served as a reference.

### 2.2 Catalyst Characterization

N_2_ physisorption isotherms were recorded on a Micromeritics TriStar II Plus apparatus at 77 K. The samples were first dried at 443 K (or at 573 K for the SiO_2_ support) under an N_2_ flow overnight. The BET surface area was determined according to the IUPAC procedure.^51^ A Barrett–Joyner–Halenda (BJH) analysis was applied to obtain pore size distributions, using either a carbon black or Harkins-Jura statistical thickness curve. The single-point total pore volume *V*_tot_ was determined at *p*/*p*_0_ = 0.995. Integration of the differential pore size distribution (derived from the adsorption branch) between 2 and 50 nm yielded the mesoporosity. The micropore volume *V*_micro_ was calculated using the *t*-plot method.

Transmission electron microscopy (TEM) imaging was performed on an FEI Tecnai 20 apparatus, operating at 200 kV. High-angle, annular, dark-field scanning transmission electron microscopy (HAADF-STEM) images were obtained on a Thermo Fisher Scientific Talos F200X apparatus, operating at 200 kV. With the same apparatus, chemical maps were recorded using energy-dispersive X-ray (EDX) detectors. The EM samples for the carbon-supported catalysts were prepared by deposition of an ethanolic dispersion of the catalyst onto holey carbon film-coated Cu or Au grids (Agar, 300 mesh). As adequate TEM measurements on the silica-supported catalysts as such were not possible, they were ultramicrotomed. The catalysts were embedded in a two-component epoxy resin (Struers, EpoFix), which was heated overnight at 333 K and cut in 60–70 nm slices on a Leica Ultracut E. The slices were deposited on the aforementioned Au grids, which were made hydrophilic by glow discharge in a Cressington 208 carbon coater. At least 350 individual particles at various locations within the sample were measured to determine the number-averaged Cu(Zn)O_*x*_ particle sizes (*d*_N_) with the standard deviation (*s*_N_) representing the width of the size distribution. These mean sizes were translated into surface-averaged particle sizes (*d*_S_) *via*, with *d*_*i*_ the *i*-th particle size and *N* the total number of measured particles. Only the relevant part of the log-normal distribution (>1% of maximum) was considered for the calculation of the average particle sizes.

Powder X-ray diffractograms were recorded on a Bruker AXS D2 Phaser diffractometer at room temperature with a fixed divergence slit. Samples were irradiated by Co Kα radiation (λ = 1.790 Å) at 30 kV and 10 mA. Not only fresh catalysts, but also used catalysts were analyzed. These were exposed to ambient conditions, separated from the SiC in the reactors, finely ground, and characterized without any further pretreatment.

Temperature-programmed reduction (TPR) profiles were obtained on a Micromeritics AutoChem II 2920 apparatus. The sample (50 mg, <75 μm granulites) was first dried *in situ* under an Ar flow at 1 L min^–1^ g_sam_^–1^ at 393 K for 30 min. The cooled sample was then exposed to 5 vol % H_2_/Ar at the same flow and heated to 873 K with a ramp of 2 K min^–1^. The formed H_2_O was captured with a dry ice/isopropanol cold trap, and the reduction profiles were recorded with a thermal conductivity detector (TCD). The H_2_ reduction profiles of the CuZn-15/C and CuZn-15/SiO_2_ catalysts (25–75 μm) were also obtained at a temperature ramp of 5 K min^–1^ in a 0.5 L min^–1^ g_sam_^–1^ flow without prior drying to directly compare with the H_2_ treatment during XAS.

Time-resolved, *operando* X-ray absorption spectroscopy (XAS) measurements on simultaneously the Cu (8979 eV) and Zn K-edges (9659 eV) were performed at the SOLEIL synchrotron (ROCK beamline).^52^ Typically, *ca.* 3.5 mg of catalyst (25–75 μm sieve fraction) was loaded in a quartz capillary (ID 1.5 mm, 50 μm thick), which was tightly glued into a frame connected to gas feed lines. Heating of the capillary was ensured by a hot gas blower (FMD Oxford). After the capillary was leak-checked at 20 bar, XAS data was obtained in He at room temperature. The catalyst was exposed to a 15 mL min^–1^ flow of 20 vol % H_2_/He and heated to 543 K (ramp 5 K min^–1^) with a hold time of 5–15 min at ambient pressure. After the H_2_ treatment, the capillary was cooled to 453 K prior to introducing a syngas feed (H_2_/CO/He = 60/30/10 vol %) at 15 mL min^–1^. Within *ca.* 100 min, the capillary was pressurized to 20 bar, and subsequently, the temperature was increased to 533 K (ramp 5 K min^–1^) and held for 160 min. Only for the CuZn-15/C catalyst was the feed subsequently switched to H_2_/CO/CO_2_/He = 60/27/3/10 vol %, recording XAS spectra for 160 min, and after that to H_2_/CO_2_/He = 67.5/22.5/10 vol %. Finally, XAS data were recorded after cooling to room temperature at 20 bar and in the last experienced gas atmosphere (H_2_/CO_2_/He and H_2_/CO/He for the CuZn-15/SiO_2_ and CuZn-15/C catalysts, respectively).

During all treatments, XAS spectra were recorded while scanning the X-ray energy from 8.70 to 10.65 keV (20 averaged scans per 10 s) in transmission mode using a Si(111) quick-XAS monochromator. Methanol production and gas compositions were recorded with a mass spectrometer (Cirrus, MKS) at ambient pressure. If the measurement involved CO, a conditioned carbonyl trap was used upstream the capillary to capture metal carbonyl compounds. ZnO (abcr, 99.999%), CuO (Sigma-Aldrich, 99.999%), Cu_2_O (Sigma-Aldrich, ≥99.99%), in-house synthesized Zn_2_SiO_4_ (Figure S27), all mixed with boron nitride (Sigma-Aldrich, 98%), and Cu (6 μm) and Zn (5 μm) foils were used as references, with their spectra being recorded at room temperature under air. A Zn_30_Cu_70_ brass measured at the ESRF (LISA beamline (BM 08)) was also used as a reference. The optics and performance at the two beamlines are different, and hence, this might give rise to slight changes in the observed XAS spectra. Data analysis was performed using Athena and Artemis software, as further detailed in supplementary section S4.

### 2.3 Catalyst Testing

A 16-reactor setup (Flowrence, Avantium) was used to evaluate the catalyst performance for methanol synthesis at 40 bar(g) and 533 K for at least 100 h. The powdered catalysts were pressed, crushed, and sieved into granules of 75–150 μm and were loaded (3–180 mg) in the stainless-steel reactors (ID 2.6 mm). The catalysts were diluted with SiC (212–245 μm fraction, Alfa Aesar, ≥98.8%, 46 grit), resulting in SiC contents between 22 and 88 vol % of the total packed catalyst bed.^53^ The SiC had been previously calcined at 1073 K for 10 h, washed with 65 wt % HNO_3_, rinsed with Milli-Q water until pH 7 was reached, and dried in static air at 393 K overnight. The varying catalyst loadings enabled us to achieve similar CO (+ CO_2_) conversions (*ca.* 10%). The difference in sieve fractions between the catalysts and diluent facilitated postanalysis by EM and XRD.

An *in situ* reduction was performed in 2.8 mL min^–1^ of 5 vol % H_2_/N_2_ at 523 K for 3 h after which the temperature was lowered to 393 K. The reactors were exposed to a 2.2 mL min^–1^ flow of CO_2_-free syngas (H_2_/CO/He = 60/30/10 vol %) or CO_2_-enriched syngas (H_2_/CO/CO_2_/He = 60/27/3/10 vol %), leading to a flow of 0.2–2.1 L min^–1^ g_Cu_^–1^ and a gas-hourly space velocity (GHSV) of 400–53 200 h^–1^. The reactors were pressurized to 40 bar(g), heated to 533 K (ramp 2 K min^–1^), and the reaction was run for at least 100 h. Alternatively, the ZnO_*x*_/C and ZnO_*x*_/SiO_2_ catalysts were alternately exposed to the predefined H_2_/CO/He and H_2_/CO/CO_2_/He feeds. A triphase carbonyl trap (activated carbon, γ-Al_2_O_3_, ZnO) was located upstream of the CO feed to remove metal carbonyls and sulfur species. Products were periodically analyzed by online gas chromatography every 15 min. After catalysis, the samples were slowly exposed to air at 393 K. Details on the calculations of activity, selectivity, and stability are given in section S5.

## 3 Results and Discussion

### 3.1 Structural Properties of the Catalysts

**Figure 1:**
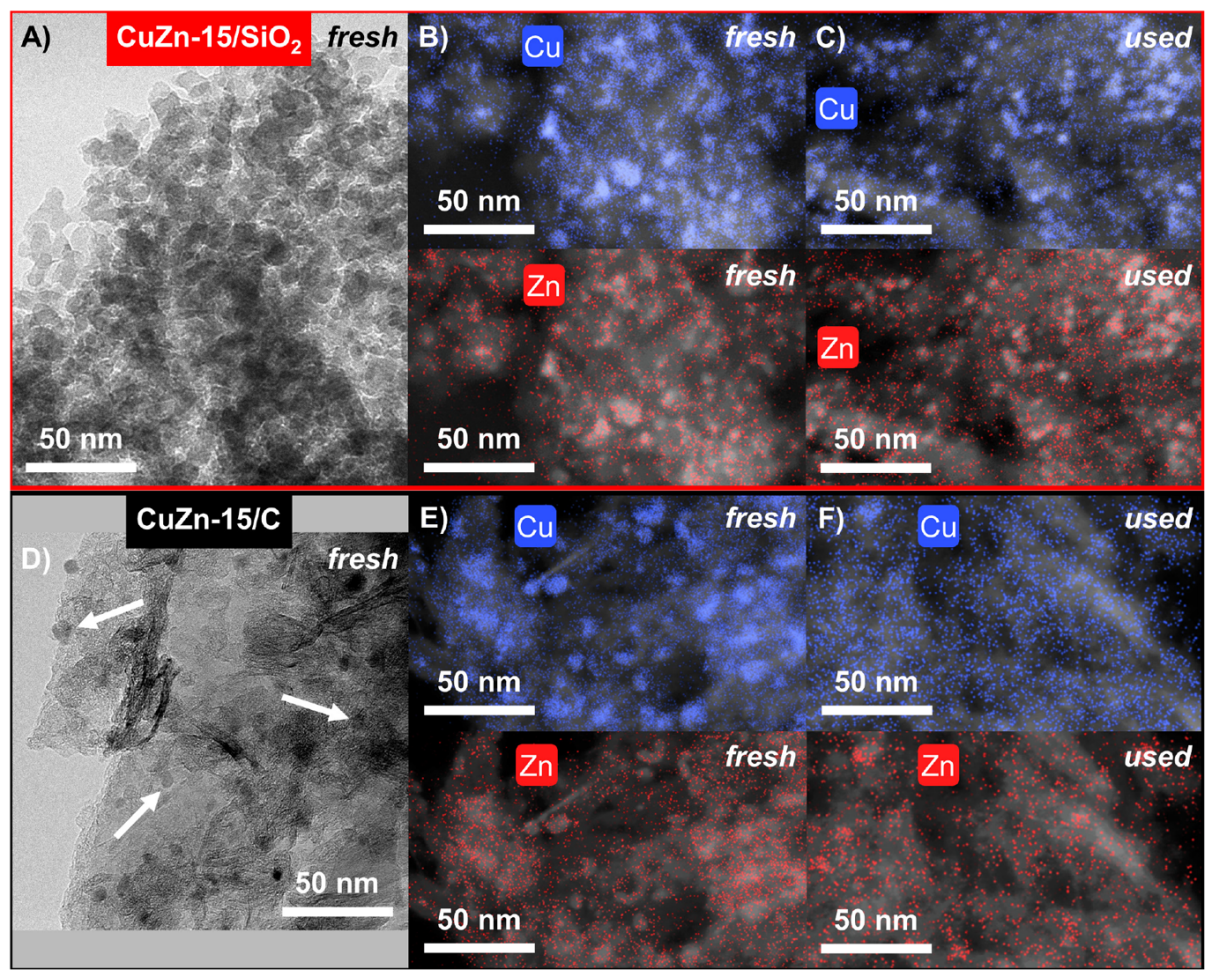
Representative EM images of the (A–C) CuZn-15/SiO_2_ and (D–F) CuZn-15/C catalysts. Frames A and D involve BF-TEM, and frames B–C and E–F involve HAADF-STEM with an elemental map overlay. Number-averaged Cu(Zn)O_*x*_ particle sizes are 3.4 ± 0.8 nm (frames B–C) and 4.2 ± 1.7 nm (frame D) for the fresh CuZn-15/SiO_2_ and CuZn-15/C catalysts, respectively. The used catalysts (frames C and F) are after 150 and 100 h of catalysis in an H_2_/CO/CO_2_ feed, respectively. Please note that the pixel size in frame F is larger (521 pm) than in frames B, C, and E (368 pm). Corresponding zoomed-out images and EDX spectra are shown in Figures S5–S6.

Figure 1 shows representative electron micrographs, including elemental maps, of CuZn-15/SiO_2_ (frames A–C) and CuZn-15/C (frames D–F) catalysts both with 8.1 wt % Cu and 1.8 wt % ZnO (see Figures S2–S5 for other catalysts and zoomed-out micrographs). Note that the number in the catalyst names refers to the Zn/(Cu + Zn) fraction of 15 at%. The silica as support consists of aggregated spheres of *ca.* 8 nm, whereas the graphitic carbon has a sheet-like morphology of a few nanometers thick. Both materials have a high specific surface area (>260 m^2^ g^–1^), and *ca.* 50–60% of the total pore volume consists of mesopores (Figure S1, Table S1), making these materials suitable supports for model catalyst studies.Frame A shows no clear Cu(Zn)O_*x*_ nanoparticles on the silica support, demonstrating that it was challenging to distinguish metal particles on the silica support because of the limited phase contrast. Only by imaging ultramicrotomed slices were we able to obtain a representative HAADF-STEM micrograph with an elemental map (frame B). Cu(Zn)O_*x*_ particles of *ca.* 3–4 nm (bright spots) were observed for the CuZn-15/SiO_2_ catalyst, corresponding to mainly Cu species (blue dots) and ZnO_*x*_ species (red dots). The distribution of Cu and ZnO_*x*_ looked similar after 150 h of catalysis (frame C), which is probably more representative for the catalyst during catalysis.

When using a graphitic support (frame D) CuO_*x*_ nanoparticles of *ca.* 4 nm were clearly discernible by TEM and well-distributed (dark spots indicated with white arrows) on the carbon surface (light gray).

The distribution was confirmed by the elemental maps of Cu and Zn species, projected on a HAADF-STEM image (frame E). There was a strong correlation between the location of the Cu nanoparticles and the distribution of the ZnO_*x*_ species, both in the fresh CuZn-15/C catalyst and after catalysis (frames E and F). The characteristics of the full series carbon-supported CuZnO_*x*_/C catalysts both in the fresh and used state can be found in the Supporting Information (Table S2) and shows similar Cu(Zn)O_*x*_ particle sizes (*d*_N_ = 5–9 nm) with varying ZnO_*x*_ loadings. Additional structural information includes N_2_ physisorption (Figure S1), additional electron microscopy imaging (Figures S2–S5), X-ray diffraction (XRD) analysis (Figure S8), and H_2_ reduction profiling (Figure S9). Overall, we showed that in both catalysts well-distributed Cu(Zn)O_*x*_ particles of similar size were present and that the relatively thin sheets of graphitic carbon as a model support facilitated the determination of the particle sizes by electron microscopy.

### 3.2 Influence of the Support and Feed Composition

**Figure 2:**
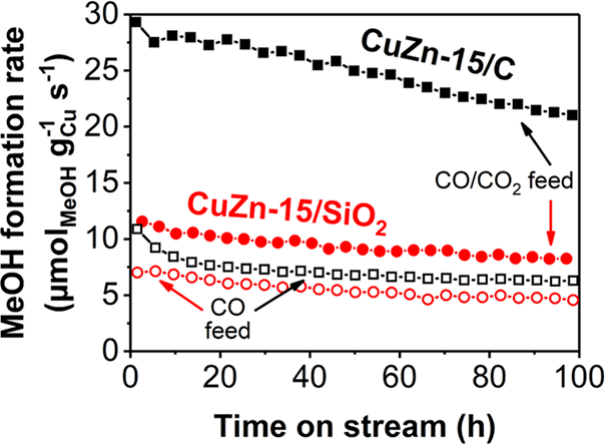
Methanol formation rate of the CuZn-15/SiO_2_ (red circles) and CuZn-15/C (black squares) catalysts in a CO_2_-free (open symbols) or -enriched (filled symbols) syngas feed. The data points of the CuZn-15/C catalyst in H_2_/CO and H_2_/CO/CO_2_ are the average over 4 and 2 separate runs, respectively. Conditions: 533 K, 40 bar(g), H_2_/CO/He = 60/30/10 vol % or H_2_/CO/CO_2_/He = 60/27/3/10 vol %.

In this section, we compare the catalytic performance of CuZn-15/SiO_2_ and CuZn-15/C catalysts, which were prepared and tested in the same way and have similar Cu(Zn)O_*x*_ particle sizes and ZnO_*x*_ loadings but only have a different support. Figure 2 shows the methanol formation rate under industrially relevant temperature and pressure as a function of time in an H_2_/CO feed as well as in an H_2_/CO/CO_2_ feed (mimicking industrially relevant conditions^1−5^). Figure S10 gives the CO (+ CO_2_) conversion and total activity, and Table S4 provides additional information on the conversion levels, turnover frequencies (TOFs), and Cu(Zn)O_*x*_ particle growth during catalysis.

The TOF for the carbon-supported catalyst (3.9–15.3 × 10^–3^ s^–1^) was always higher than for the silica-supported catalyst (1.6–3.0 × 10^–3^ s^–1^) (Table S4). Strikingly, the beneficial effect of CO_2_ enrichment of the syngas feed on the methanol formation rate was much larger for the CuZn-15/C catalyst (factor 3.5) than for the CuZn-15/SiO_2_ catalyst (factor 1.7) (Figure 2). Upon CO_2_ enrichment the methanol selectivity increased from 83 to 99%_C_ and from 85 to 98%_C_ after 100 h on stream for, respectively, the CuZn-15/SiO_2_ and CuZn-15/C catalysts, in line with earlier published results^5^ and significantly higher than recently reported for CuZnO_*x*_/Al_2_O_3_.^24^ In the literature, enhancement factors upon CO_2_ enrichment of 2–4 are reported for Cu/ZnO/Al_2_O_3_ catalysts depending on the reaction conditions,^2,3,47^ and differences were also observed between silica- and alumina-supported CuZnO_*x*_ particles.^24^ However, our results, obtained in the same reaction conditions and with similar Cu particles sizes, unequivocally proves that promotion with a given amount of ZnO_*x*_ is much more efficient using a carbon than using an oxide support. Under all conditions, the ZnO_*x*_ promotion is more effective in the CuZn-15/C catalyst than in the CuZn-15/SiO_2_ catalyst, but the effect is especially pronounced with CO_2_ enrichment of the feed.

**Figure 3:**
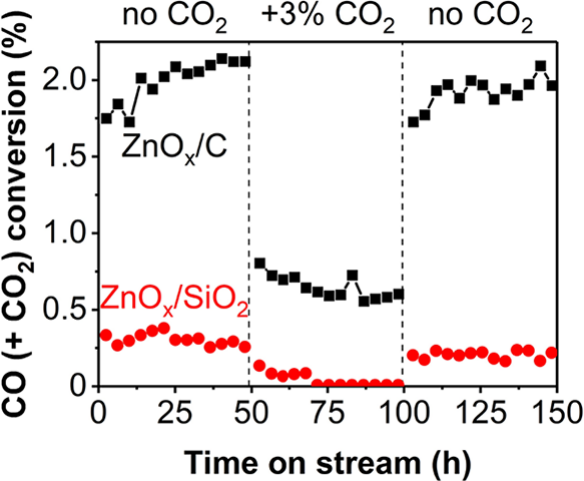
CO (+ CO_2_) conversion of silica- and carbon-supported ZnO_*x*_ (10 wt %) in various syngas compositions. Label “+3% CO_2_” in the total feed corresponds to a CO_2_/(CO + CO_2_) volume fraction of 0.10. Conditions: 533 K, 40 bar(g), H_2_/(CO + CO_2_)/He = 60/30/10 vol %, 21.9 mL min^–1^ g_cat_^–1^.

It is known that ZnO_*x*_ itself can also act as a methanol synthesis catalyst, albeit with a lower activity than in combination with Cu.^16,54,55^ Supported ZnO_*x*_ species without Cu were investigated under similar reaction conditions to check if the catalysis by ZnO_*x*_ on graphitic carbon contributed significantly. The ZnO_*x*_/SiO_2_ and ZnO_*x*_/C catalysts have the same ZnO loading (both 10 wt %) with ZnO_*x*_ particle sizes of 7.7 and *ca.* 4.5 nm, respectively. Figure 3 shows the CO (+ CO_2_) conversion versus time on stream in the presence and absence of CO_2_ and for both an oxidic and a carbon support.

Irrespective of the syngas composition, carbon-supported ZnO_*x*_ species were much more active than silica-supported ZnO_*x*_ species, even when taking the slightly different Zn surface areas into account (Figure S12). EM analysis showed no Zn-based nanoparticles (Figure S4) in the fresh ZnO_*x*_/SiO_2_ catalyst (frame B), whereas they were present in the ZnO_*x*_/C catalyst (frame A). During pure CO hydrogenation, the ZnO_*x*_/C catalyst had a significant conversion of *ca.* 2% (of which *ca.* 1.3% was methanol (Figure S12)). The activity of both supported ZnO_*x*_ species clearly decreased in the presence of CO_2_. The conversion level was restored when switching back to an H_2_/CO feed. This demonstrates that the negative CO_2_ effect on the conversion is not related to, for example, irreversible changes in the catalyst morphology but probably can be attributed to the significant reduction of ZnO in a CO_2_-free feed, making it a more efficient methanol synthesis catalyst. Yet, the activity of the supported ZnO_*x*_ was too small to explain the overall effects of CO_2_-enrichment in methanol synthesis of CuZnO_*x*_-based catalysts. Nevertheless, these results clearly show that an oxide support has a strong interaction with the ZnO_*x*_ and leads to a different speciation than for the weaker interacting carbon supports.

### 3.3 Influence of the ZnO_*x*_ Loading on Activity and Stability

**Figure 4:**
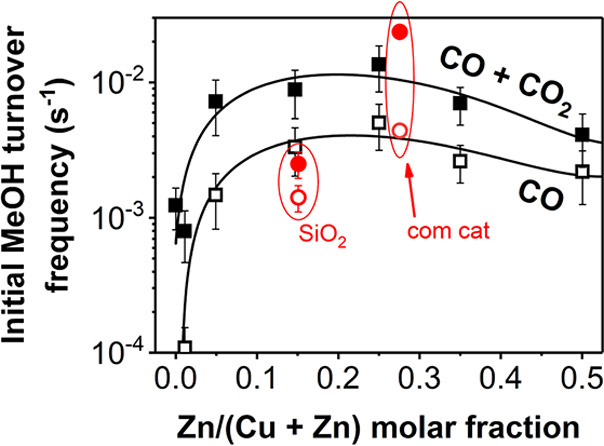
Initial methanol turnover frequency (TOF_MeOH_) of CuZnO_*x*_/C (black squares) and metal oxide-supported (red circles) catalysts in an H_2_/CO (open symbols) or an H_2_/CO/CO_2_ (filled symbols) feed (at *t* = 0). “SiO_2_” = CuZn-15/SiO_2_ catalyst. “com cat” = commercial, coprecipitated Cu/ZnO/Al_2_O_3_/MgO catalyst (58 wt % Cu, *ca.* 10 nm CuO particles). Conditions: 533 K, 40 bar(g), H_2_/CO/He = 60/30/10 vol % or H_2_/CO/CO_2_/He = 60/23/7/10 vol %.

For the carbon-supported catalysts, we investigated in more detail the effect of the ZnO_*x*_ loading on the activity and stability of supported Cu nanoparticles. Figure 4 shows the specific activity (see Figures S13–S14 for the total activity) as a function of the ZnO_*x*_ loading in a syngas atmosphere with and without CO_2_ (please note the logarithmic scale) for *ca.* 5.1 nm Cu nanoparticles supported on either a carbon support (black lines) or an oxide support (red markers). The activity increased when CO_2_ was in the feed for all studied ZnO_*x*_-promoted catalysts. After the initiation period, all catalysts had a methanol selectivity of >97%_C_ in CO_2_-enriched syngas. The highest TOF_MeOH_ values were obtained for carbon-supported catalysts with Zn/(Cu + Zn) molar fractions between 0.15 and 0.25, irrespective of the presence of CO_2_ in the feed. These ZnO_*x*_ loadings are lower than the well-established optimal loading for the commercially used Cu/ZnO/Al_2_O_3_/MgO methanol synthesis catalyst as well as for other oxide-based Cu catalysts in literature (Zn/(Cu + Zn) content of 29–47 at%).^10,13,15,19,22,23,28−31,56^

Another important factor in catalysis is the stability. In Figure S15, this stability is defined as the ratio between the activity after 100 h and after 50 h on stream. The addition of only 5 at% ZnO_*x*_ was sufficient to increase the catalyst stability from 74 ± 8% to 84 ± 3% upon syngas conversion. Further increasing the ZnO_*x*_ content to 15–35 at% maximized the stability to 91 ± 2% and 83 ± 3% in an H_2_/CO and H_2_/CO/CO_2_ feed, respectively. In the most heavily promoted CuZn-50/C catalyst, the stability was somewhat lower. The presence of 15–35 at% ZnO_*x*_ apparently limited the CuZnO_*x*_ particle growth during catalysis as evident from TEM and XRD analysis (Figures S3, S7, and S8) and is in line with the stability improvement for intermediate amounts of ZnO_*x*_. Hence, ZnO_*x*_ is not only an activity promoter but also a stability promoter for carbon-supported Cu catalysts.

### 3.4 Catalyst Evolution during Reduction in H_2_

From the literature, it is known that the coverage of the Cu surface with ZnO_*x*_ species^13^ and the reduction degree of these ZnO_*x*_ species^14,35^ are parameters that determine the effectiveness of ZnO_*x*_ as a promoter. However, mostly metal oxides are employed to support CuZnO_*x*_ particles, which can result in the formation of spectator species such as zinc silicates and aluminates,^11,37,43^ hampering the study of the active fraction of the ZnO_*x*_ promoter. The presence of the mixed Zn metal oxides may hence obscure the results also of, for example, electron energy loss spectroscopy (EELS) and *operando* X-ray absorption spectroscopy (XAS) measurements. While EELS is a valuable technique to study the local oxidation state of metals,^56,57^ we chose to assess the chemical state of our supported catalysts by XAS because of the small particle sizes and relatively low metal loadings. Hence, our hypothesis was that our use of a carbon support would allow us to much better study the formation, oxidation state, and structure of the relevant ZnO_*x*_ promoter by time-resolved, *operando* XAS at simultaneously the Cu and Zn K-edges at 20 bar and up to 533 K.

**Figure 5:**
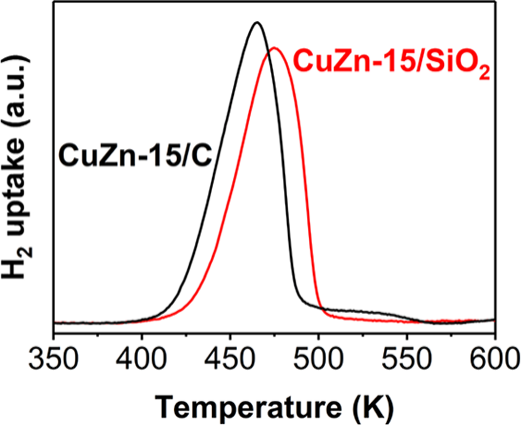
*Ex situ* reduction in 0.5 L min^–1^ g_cat_^–1^ flow of 5 vol % H_2_/Ar at 5 K min^–1^ in 1 bar, mimicking the conditions used during *in situ* H_2_ treatment monitored by XAS.

A first piece of information about the interaction between CuO_*x*_ and ZnO_*x*_ species can be derived from the reduction profiles. Figure 5 shows the *ex situ* H_2_ reduction profiles of the CuZn-15/SiO_2_ and CuZn-15/C catalysts. The theoretically maximum Cu surface coverage by a monolayer of Zn atoms is 75–95% for these catalysts with 15 at% ZnO_*x*_. The maximum CuO reduction temperature (*T*_max_) as well as the offset temperature for reduction (*T*_offset_) were clearly lower for the CuZn-15/C catalyst than for the CuZn-15/SiO_2_ catalyst (*T*_max_ of 465 *vs* 475 K, *T*_offset_ of 421 *vs* 434 K, respectively). Hence, the CuO is more easily reduced on a carbon support than on a silica support. We ascribe this to a stronger interaction of CuO_*x*_ with silica.

The reducibility of the CuZn-15/SiO_2_ and CuZn-15/C catalysts was also investigated with *in situ* XAS under similar conditions as for the *ex situ* H_2_ treatments. Time-resolved X-ray absorption spectra (Figure S16) were analyzed by fitting linear combinations of the macrocrystalline references to extract the Cu oxidation state evolutions (Figure S17), showing that the CuO species in both catalysts were fully reduced to Cu^0^*via* the formation of Cu^+^ upon an H_2_ treatment up to 543 K for 5–15 min. This was confirmed by a more in-depth study using multivariate analysis in which no prior information on the component spectra was imposed but which yielded eigenspectra that corresponded well to the macrocrystalline Cu references (Figure S18). This full reduction of CuO in ZnO_*x*_-promoted CuO nanoparticles has also been reported in the literature.^14,24,42,58^

**Figure 6:**
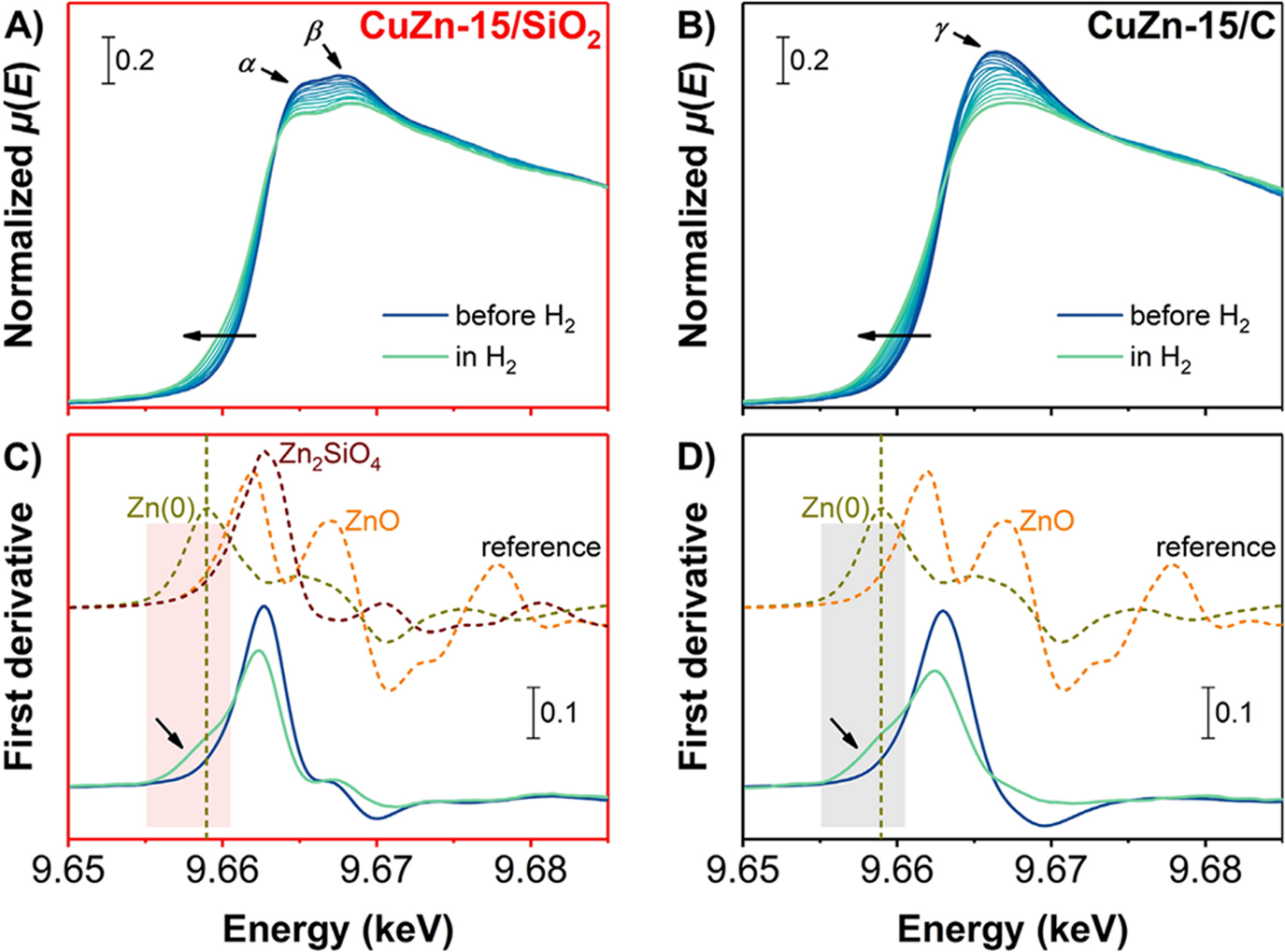
(A,B) Time-resolved, normalized absorption and (C,D) corresponding first derivatives of *in situ* XANES spectra at the Zn K-edge of the (A,C) CuZn-15/SiO_2_ and (B,D) CuZn-15/C catalysts (solid lines). The spectra are depicted in the initial state at 298 K, during a treatment in 20 vol % H_2_/He up to 543 K in 1 bar each *ca.* 5.7 min, and finally in an H_2_ atmosphere at 453 K. Dashed lines show the first derivatives of macrocrystalline ZnO, Zn_2_SiO_4_, and Zn foil references at 298 K.

During the H_2_ treatment we also studied changes in the ZnO_*x*_ oxidation state by *in situ* XAS. Figure 6 presents the time-resolved, normalized X-ray absorption near edge structures (XANES) and first derivatives at the Zn K-edge before and upon the H_2_ treatment. We start with ZnO_*x*_ species in the Zn(II) oxidation state for both CuZn-15/SiO_2_ (frames A and C) and CuZn-15/C (frames B and D) catalysts, as clear from the comparison to the first derivative of the ZnO reference. Upon heating in an H_2_ atmosphere the Zn K-edge shifted to a lower energy (indicated by the arrows), showing that partially reduced ZnO_*x*_ was formed in both catalysts. The dominant features were still due to the presence of Zn^2+^, as clear from the peak at 9.6626 keV on the first derivatives, although its intensity had slightly decreased.

Interestingly, the CuZn-15/SiO_2_ catalyst (frame A) displayed two distinct peaks in the normalized XANES spectra (indicated with α and β). This peak combination has been reported before and is ascribed to the presence of zinc silicates in a single phase such as Zn_2_SiO_4_.^37,59−62^ Yet, the CuZn-15/C catalyst (frame B) only had one single, broad peak (indicated with γ), in line with a ZnO_*x*_ phase which was also observed in electron microscopy (Figure 1, frame E). The estimated, average Zn oxidation number (ON) was slightly lower for the CuZn-15/C catalyst than for the CuZn-15/SiO_2_ catalyst (+1.3 *vs* +1.6, see also Table S5). Multivariate analysis on the Zn K-edge is more challenging than for the Cu K-edge, as the XAS signal is lower. Extraction of the components (Figure S20) suggests the presence of three distinct phases for the CuZn-15/SiO_2_ catalyst. The eigenspectrum of one of the components resembles that of Zn_2_SiO_4_ and its contribution is relatively stable throughout the experiment, indicating the presence of a substantial amount of Zn spectator species in the CuZn-15/SiO_2_ catalyst. It has to be noted that because of the relatively low Zn loading, these spectator species were not observed by infrared spectroscopy (Figure S26). For the carbon-supported catalyst, a significant contribution of a compound with a relatively high absorption at lower energies is found (Figure S20). The phases do not fully match with the macrocrystalline Zn references, which indicates highly dispersed species of low crystallinity and/or not very well-defined mixed phases. This confirms the impact of the support on the ZnO_*x*_ speciation: on an oxidic support, the majority of the Zn species is irreducibly bound to the oxidic support and a fraction of the Zn is bound in silicate species, while on a carbon support a highly dispersed ZnO_*x*_ phase with an average Zn oxidation number significantly lower than +2 is present, which might be due to a high defect density in the ZnO (creating oxygen vacancies and a lower average ZnO state) or possibly the intermixing of fully reduced Zn in the compounds.

### 3.5 Nature of the ZnO_*x*_ under Working Conditions

**Figure 7:**
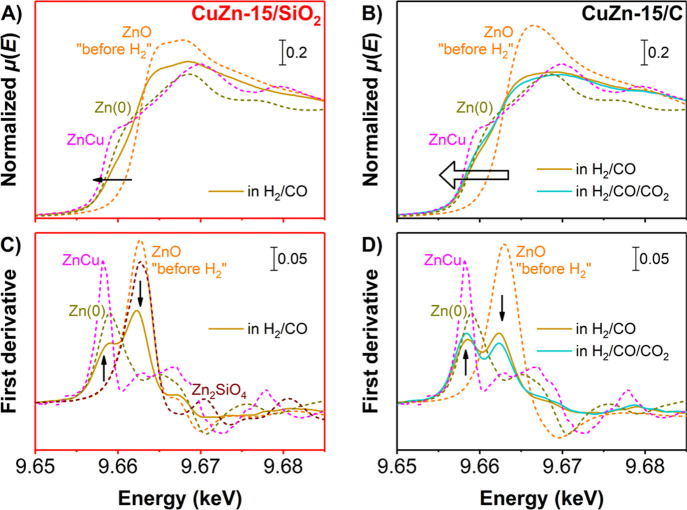
(A,B) Normalized absorption and (C,D) corresponding first derivatives of *operando*, normalized XANES spectra at the Zn K-edge of the (A,C) CuZn-15/SiO_2_ and (B,D) CuZn-15/C catalysts (solid lines). Depicted during H_2_/CO (and subsequent H_2_/CO/CO_2_) conversion at 20 bar and 533 K, each after 160 min. Gas compositions: H_2_/CO/He = 60/30/10 vol % and H_2_/CO/CO_2_/He = 60/27/3/10 vol %. Dashed lines show the initial catalyst state (ZnO), macrocrystalline Zn_2_SiO_4_ and Zn_30_Cu_70_, and Zn foil at 298 K.

The *in situ* H_2_-treated catalysts were used for high-pressure methanol synthesis by CO, CO/CO_2_, and CO_2_ hydrogenation. Upon catalysis, no significant changes in the oxidation state and local coordination of the Cu^0^ were detected (Figure S21), in line with results published earlier.^39,45^Figure 7 shows the normalized, *operando* XAS spectra of the Zn K-edge in the XANES region after 160 min in an H_2_/CO feed and after 160 min of subsequent H_2_/CO/CO_2_ feed for the CuZn-15/SiO_2_ (frames A and C) and CuZn-15/C (frames B and D) catalysts. The overall results, including CO_2_ hydrogenation, are shown in Figures S19 and S21–S24. Note that because of the XAS setup restrictions, the amount of catalyst and hence the conversion was limited (Figure S25). For the CuZn-15/SiO_2_ catalyst, the ZnO_*x*_ was only slightly further reduced during methanol synthesis (frames A and C) with an estimated Zn ON of +1.1. This was confirmed by a measurement after cooling the catalyst down to room temperature to obtain sharper features (average Zn ON of +1.2, Figure S19). Features that were attributed to zinc silicates were dominant at all stages in the XAS spectra for the CuZn-15/SiO_2_ catalyst, as confirmed by multivariate analysis (Figure S20). The fact that only a slight reduction of the Zn(II) is observed when using oxidic supports and that the Zn species strongly interact with the support is in line with earlier reports using oxidic supports.^37,45^

Remarkably, in the CuZn-15/C catalyst, a large fraction of metallic Zn was formed during methanol synthesis at 20 bar (Figure 7, frames B and D). This observation was confirmed by multivariate analysis, which showed a resemblance of the independently extracted eigenspectrum of the Zn species to the XAS spectrum of metallic Zn (Figure S20). The average Zn ON was only *ca.* + 0.6. Assuming that the Zn species are either in the Zn(II) or Zn(0) oxidation state, this means that about 70% of the Zn species was completely reduced. With the addition of 3 vol % CO_2_ in the feed, a slightly less-reducing gas atmosphere was created. Nevertheless, the Zn ON decreased further with time to *ca.* + 0.5 after nearly 3 h in the H_2_/CO/CO_2_ feed, which is probably rather an effect of time than feed composition. Upon switching to a pure H_2_/CO_2_ feed, the average Zn ON slightly increased to + (0.6–0.8). An increase is expected in a more oxidizing gas feed, as it is also predicted computationally that there will be a slight dependence of the ON (and hence probably the Zn coverage) on the exact feed composition.^13^ However, even under these conditions, most of the Zn species remain in the fully reduced state under *operando* conditions. This means that the relatively high Zn ONs in methanol synthesis systems reported until now (in the presence of an oxidic support) can probably be explained by a strong promoter–support interaction, and hence, a large fraction of the Zn promoter species is being chemically bound to the oxidic support (and hence inactive). In contrast, our use of a weakly interacting carbon support allows us to assess an average Zn oxidation state and coordination number that are much more representative of the true nature of the active ZnO_*x*_ promoter phase during methanol synthesis.

Several hypotheses have been postulated for the ZnO_*x*_ speciation during Cu-catalyzed methanol synthesis. First, the beneficial effect of the ZnO_*x*_ promoter was ascribed to the so-called strong metal–support interaction (SMSI) with slightly reduced ZnO having a high affinity for the Cu^0^ metal and partially covering the Cu nanoparticle surface.^35,63^ Alternative explanations involve the influence of ZnO_*x*_ on the structure of the Cu nanoparticles. For instance, it was proposed that specific steps sites were exceptionally active sites on the Cu^0^ surface and that these step sites were stabilized by Zn^0^ atoms.^10,39,64^ Metallic Cu and Zn are quite miscible; up to 33 at% Zn can dissolve in Cu (solid solution) at temperatures between 473 K and the melting point (>1175 K).^65^ Some groups proposed that the active site was related to the decoration of Cu^0^ nanoparticles with Zn^0^ atoms and shallow diffusion of Zn^0^ atoms into the Cu surface.^13,21,44^ However, results from *ex situ* and low-pressure studies have limited value, as it is known that the catalytically active phase dynamically adjusts to the working conditions.^66^ A recent high-pressure *operando* study, based on oxidic supports, concluded that a distorted ZnO_*x*_ layer was the majority phase under working conditions with at most 9% of the Zn being present as Zn^0^ atoms.^24^ Our experiments clearly show that, if a strong interaction of the Zn species with an oxide support is avoided, a much more truthful picture of the active fraction of the Zn promoter species under high-pressure methanol synthesis conditions is obtained, and that this fraction is clearly reduced to zerovalent Zn upon prolonged methanol synthesis conditions (Figure 7).

**Figure 8:**
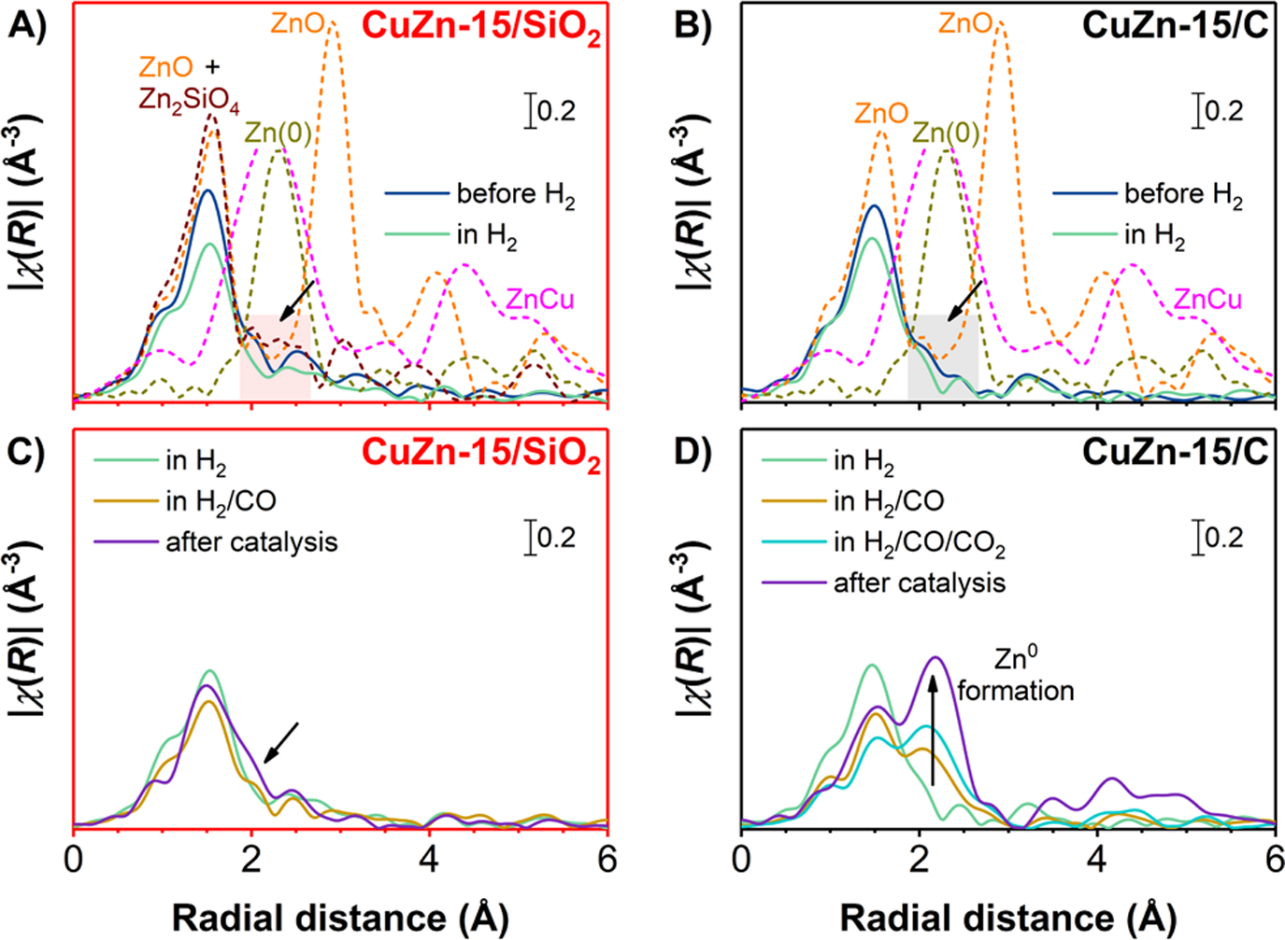
Fourier-transformed EXAFS spectra at the Zn K-edge of the (A,C) CuZn-15/SiO_2_ and (B,D) CuZn-15/C catalysts (solid lines). (A,B) Depicted during *in situ* reduction in the initial state at 298 K and in an H_2_ atmosphere at 453 K after an H_2_ treatment at 1 bar (for conditions, see Figure 6). (C,D) Depicted during H_2_/CO (and subsequent H_2_/CO/CO_2_) conversion at 533 K and 20 bar (for conditions, see Figure 7) and after catalysis. Dashed lines depict the macrocrystalline ZnO, Zn_2_SiO_4_, Zn_30_Cu_70_, and Zn foil references. The unlabeled arrows indicate the position of Zn–Zn or Zn–Cu bond formation.

Zooming in on the local coordination of the Cu and Zn atoms during high-pressure methanol synthesis, we analyzed the extended X-ray absorption fine structure (EXAFS) region of the XAS data. Figure 8 shows the EXAFS data on the Zn K-edge in *R*-space for the CuZn-15/SiO_2_ (frame A) and CuZn-15/C (frame B) catalysts in the initial state and upon heating in an H_2_ atmosphere. The EXAFS-derived *R*-spaces at the Cu K-edge and the EXAFS fitting parameters are available in Figure S24 and Tables S6–S9, respectively. The initial spectra of both catalysts have a main peak at 1.50 Å in the Fourier transform, which corresponds to first-shell Zn–O bonds such as in ZnO with a bond length of 1.97 Å. The *R*-space of the CuZn-15/SiO_2_ catalyst (frame A) closely resembles that of the Zn_2_SiO_4_ reference, showing that a majority of the Zn atoms is bound to the oxide support, as reported before.^37^ No contribution of second-shell Zn–Zn bonds was observed (frames A and B) (which could be expected in crystalline ZnO at 2.91 Å in the nonphase corrected Fourier transform, corresponding to a real bond length of of 3.2 Å^41^), indicating the absence of larger ZnO crystallites in both samples and in line with the high ZnO_*x*_ dispersion observed by TEM (Figure 1). During *in situ* H_2_ reduction, the Zn–O bond intensity at 1.50 Å apparently decreased for both catalysts, but this was simply due to the increasing measurement temperature^35^ as the overall peak intensities significantly increased in the spectra taken at room temperature after catalysis (purple lines in frames C and D) compared with the spectra taken during the last stage of catalysis at high temperature. It is important to note that in neither of the catalysts was Zn^0^ formation observed during reduction in atmospheric-pressure H_2_ (the Zn–Zn or Zn–Cu bond fingerprint is expected at 2.30 Å in the nonphase corrected Fourier transform, its position is indicated with an unlabeled arrow in the frames). After the *in situ* H_2_ reduction, the first-shell Cu–Cu coordination number (CN) in metallic Cu was *ca.* 11 (for bulk Cu^0^ this CN is 12), and it remained unchanged for both catalysts, independent of the exact feed. This means that no change in the Cu nanoparticles was observed upon exposure to working conditions: neither a significant fraction of oxidized copper nor the presence of highly dispersed copper. This is in agreement with the fully reduced Cu observed in the spectra at the XANES region.

We continue the EXAFS analysis under *operando* methanol synthesis conditions by focusing on the Zn local surrounding. Figure 8 (frames C,D) shows selected *R*-spaces from the EXAFS data on the Zn K-edge for both catalysts (for the complete set, please see Figure S23). For the CuZn-15/SiO_2_ catalyst during CO hydrogenation, only a very minor fraction of metallic Zn was observed (signal around 2.30 Å indicated with the arrow in frame C), in line with earlier reports on oxide-supported catalysts.^24,35,63^ Nevertheless, a change was observed, as the first-shell Zn–O CN decreased from 4 (as in bulk ZnO and Zn_2_SiO_4_) to 2.6 ± 0.5 and a very low second-shell Zn–Zn or Zn–Cu (from here onward denoted as Zn–M) CN of 2.2 ± 1.4 was obtained (for bulk Zn^0^ this CN is 12). This indicates a very slight change in the average Zn surroundings, but because of the small changes and the very similar Zn–Zn and Zn–Cu bonding distances, it is not possible to analyze this in detail. Overall, the signal remains dominated by features that are attributed to Zn silicate species, and there is very little difference between the reduced fresh catalyst and that under working conditions.

Interestingly, the CuZn-15/C catalyst (frame D) displayed large changes when switching to working conditions, which was already expected from the zerovalent Zn as evidenced by the XANES analysis (Figure 7, frame B). An average Zn–M bond length of 2.54 Å (close to that of 2.66 Å of the Zn^0^ foil reference) and a quite high Zn–M CN of 6.1 ± 1.3 were obtained. This is a clear supporting evidence for the large fraction of zerovalent Zn species in the active catalysts. Upon prolonged exposure (while slightly enriching the feed with CO_2_), the increase in coordination number continues to a Zn^0^ CN of *ca.* 8. This means that the majority of the Zn promoter species is present in metal nanoparticles. The very similar Zn–Zn and Zn–Cu bonding distances do not allow us to unequivocally derive the nature of these metal nanoparticles. However, the zerovalent Zn is very likely located in Cu–Zn nanoparticles. The high Zn–M coordination number suggests that the Zn does not remain as adatoms or a monolayer on the outside of the Cu particle. The diffusion coefficient of Zn^0^ in Cu^0^ strongly depends on the Cu particle size and temperature (see also Table S10)^67−69^ but is high enough to support a full distribution of the Zn^0^ throughout the relatively small Cu^0^ particles at the time scale of hours, in line with the XRD pattern of the used CuZn-15/C catalyst showing a small downshift of the Cu^0^ diffraction line and hence suggests CuZn alloy formation (Figure S8, frame D).

Interestingly, the time scale of the formation of highly coordinated zerovalent Zn is quite in line with the generally observed activation period for Cu methanol synthesis catalysts exposed to high-pressure working conditions.^5,9^ To our knowledge, no clear explanation for this activation period has so far been reported in academic literature, but our results suggest that the gradual reduction of Zn(II) to active Zn(0) promoter species might be an important factor in this activation.

**Figure 9:**
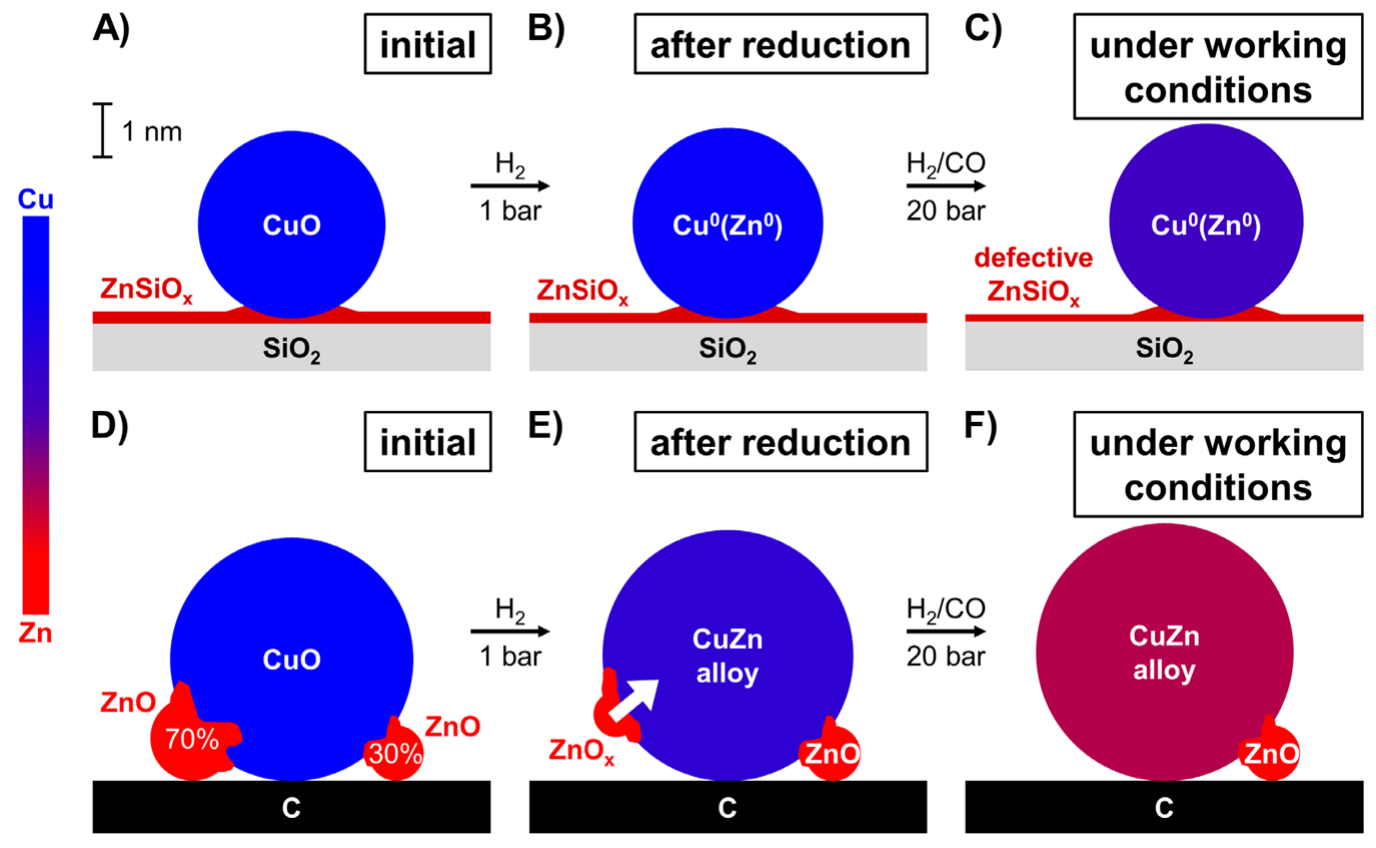
Schematic representation of the ZnO_*x*_ speciation in the (A-C) CuZn-15/SiO_2_ and (D-F) CuZn-15/C catalysts, depicted (A,D) in the initial state, (B,E) after reduction, and (C,F) under working conditions at 20 bar and 533 K. The various shades of between blue (Cu) and red (Zn) in the CuZn particles represent the relative extent of Zn^0^ incorporation into the Cu^0^ nanoparticles based on the estimated Zn ONs from the XANES analysis. For frames B and C, separate Cu^0^ nanoparticles may exist next to alloyed CuZn particles.

Figure 9 summarizes the results of our study by depicting the ZnO_*x*_ speciation in silica- and carbon-supported Cu catalysts containing 15 at% Zn/(Cu + Zn) after reduction as well as during high-pressure methanol synthesis. On both supports, the Cu^2+^ nanoparticles (depicted in dark blue) were fully reduced to Cu^0^ nanoparticles in 5–15 min exposure to 1 bar H_2_ at 543 K. Even during high-pressure methanol synthesis with a H_2_/CO_2_ feed, no significant subsequent change in the oxidation state of the Cu was observed. Using an oxidic support, which is standard in commercial catalysts and most academic studies, it was difficult to derive detailed information about the speciation of the Zn component (depicted in red) that was active as a promoter. Only slight changes in the average Zn speciation were observed (in line with earlier literature), as the signal was dominated by Zn species that had a strong interaction with the oxidic support (depicted as a layer of ZnSiO_*x*_), and these species remained dominant under all (also methanol synthesis) conditions. In contrast, using a much less strongly interacting carbon support, allowed us to follow the fraction of the Zn species that was closely affiliated with the Cu nanoparticles and hence most likely represents the active Zn promoter species during catalysts. Under methanol synthesis conditions, the relevant ZnO_*x*_ phase is in a deeply reduced state with an average Zn oxidation number of only +0.6. Assuming that only Zn^2+^ and Zn^0^ species exist, this means that *ca.* 70% of the ZnO is fully reduced to Zn^0^. The Zn–M coordination number was as high as 8 during methanol synthesis working conditions, showing that the Zn^0^ is almost fully coordinated with other metal atoms and has likely mostly diffused into the Cu nanoparticles. It is likely that this Zn speciation for the active promoter species is also relevant for the more conventional oxide-supported catalysts, to which much more Zn must be added to reach an optimum promoter effect, which is probably explained by the fact that a large fraction of the added Zn is not active as promoter.

## 4 Conclusions

Cu nanoparticulate catalysts on graphitic carbon were prepared, and compared to SiO_2_-supported catalysts, to better understand the interaction between the Cu and the Zn-based promoter species and the speciation of Zn acting as a promoter during high pressure methanol synthesis. With a modest amount of ZnO_*x*_ promoter, the methanol formation for the CuZnO_*x*_/C catalyst was significantly faster than for a CuZnO_*x*_/SiO_2_ catalyst with similar Cu particle size in a pure H_2_/CO feed. This difference was even much more pronounced in a CO_2_-enriched syngas feed. Importantly, the use of graphitic carbon model supports allowed us to reveal the true speciation of the active fraction of the Zn-based promoter under commercially relevant methanol synthesis conditions. The vast majority of the Zn(II) is reduced all the way to Zn(0) during methanol synthesis at 20 bar. Also the Zn coordination number was high, making it likely that the Zn(0) diffused into the Cu nanoparticles. The characteristic time for this diffusion corresponds to the activation time that is generally observed with this type of catalysts. For the first time this gives direct insight into the nature of the active fraction of the Zn-based promoter in high pressure methanol synthesis, not obscured by the commonly large fraction of Zn species that strongly interacts with an oxidic support, and hence dominates the structural characterization results.

## Supporting Information Available

Brief descriptions of electron imaging, crystallinity, reducibility, and the porosity of the catalysts; additional information on the catalyst activity and stability; complete set of XAS data; and details on the calculation of catalyst performance (PDF)
