# Supplementary material for: Insight into the Nature of the ZnOx Promoter during Methanol Synthesis
Source: ACS Catal. 2022 May 20;12(11):6628–39. doi: 10.1021/acscatal.1c05101 (PMC9171830; doi:10.1021/acscatal.1c05101)
Supplement: Supplementary file 1 — cs1c05101_si_001.pdf [file cs1c05101_si_001.pdf]

SUPPLEMENTARY INFORMATION

Insight into the nature of the ZnO_x promoter during methanol synthesis

Remco Dalebout[1], Laura Barberis[1], Giorgio Totarella[1], Savannah J. Turner[1], Camille La Fontaine[2], Frank M.F. de Groot[1], Xavier Carrier[3], Ad M.J. van der Eerden[1], Florian Meirer[4], and Petra E. de Jongh[1]*

[1] *Materials Chemistry and Catalysis, Debye Institute for Nanomaterials Science, Utrecht University, Universiteitsweg 99, 3584 CG, Utrecht, The Netherlands.*

[2] *Synchrotron SOLEIL, L'Orme des Merisiers, Saint-Aubin BP 48, Gif-sur-Yvette Cedex, 91192, France.*

[3] *Laboratoire de Réactivité de Surface, UMR CNRS 7197, Sorbonne Université, 4 place Jussieu, Paris Cedex 05, 75252, France.*

[4] *Inorganic Chemistry and Catalysis, Debye Institute for Nanomaterials Science, Utrecht University, Universiteitsweg 99, 3584 CG, Utrecht, The Netherlands.*

**Corresponding author: P.E.deJongh@uu.nl.*

Table of contents

Section S1: TEM imaging, crystallinity, reducibility, and porosity of catalysts

Section S2: Catalytic activity

Section S3: Catalyst stability

Section S4: X-ray absorption spectroscopy

Section S5: Calculation of catalytic activity, selectivity, and stability

Section S1: TEM imaging, crystallinity, reducibility, and porosity of catalysts

Figure S1 and table S1 present N_2 physisorption data of the pristine silica and carbon support as well as the fresh ZnO_x/SiO_2 and ZnO_x/C catalysts. Figures S2-4 show representative TEM micrographs of all fresh and, where applicable, used catalysts. All images are accompanied with particle size histograms. Figures S6-S7 present chemical mapping of selected fresh and used catalysts, including EDX spectra. Figure S8 shows all XRD measurements of fresh and used catalysts, and figure S9 shows the reducibility of the fresh catalysts in an H_2 atmosphere. Table S2 summarizes all average particle sizes. Finally, a note on the catalyst synthesis and reported metal loadings is given (table S3). For additional information on the commercial $Cu/ZnO/Al_2O_3/MgO$ catalyst (com cat) we refer the reader to our previous publication.[1]

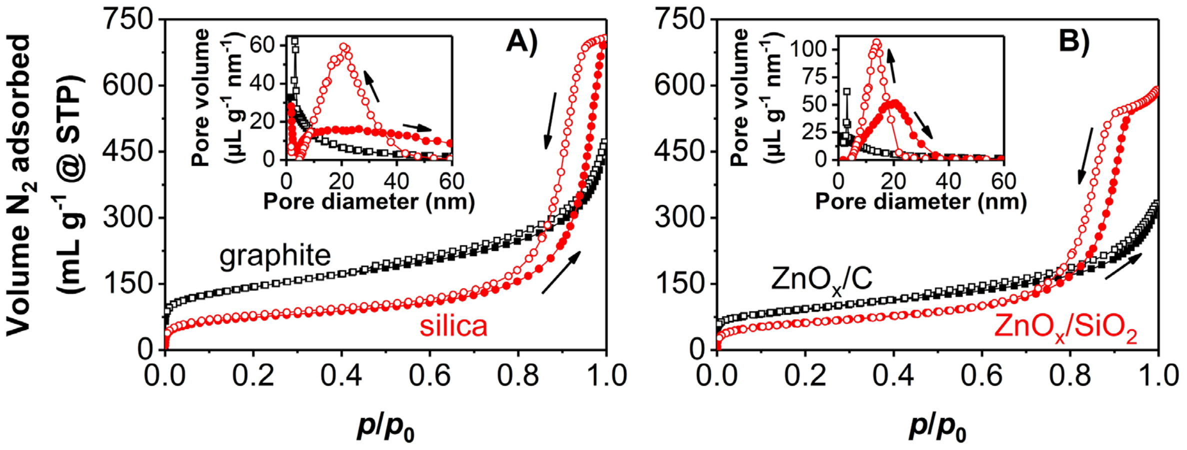

Figure S1 *N_2 physisorption isotherms of (**A**) the silica and graphite supports and (**B**) the fresh ZnO_x/SiO_2 and ZnO_x/C catalysts. Insets = number-based pore size distribution. The ZnO_x/SiO_2 catalyst has a decreased S_{BET} and V_{tot} of* ca. *15% and a decreased average pore size from 21 to 14 nm compared to the silica support. The pore morphology and distribution did not change for the ZnO_x/C catalyst compared to the graphite support.*

Table S1 *Porosity properties of the pristine supports and supported ZnO_x catalysts by N_2 physisorption.*

parameter	silica	graphite	ZnO_x/SiO_2	ZnO_x/C
BET (m^2 g^{-1})	269.3	509.2	226.6	326.1
c value	141	295	52	291
V_{micro} (mL g^{-1})	0.020	0.045	0.011	0.030
V_{meso} (mL g^{-1})	0.65	0.36	0.90	0.27
V_{tot} (mL g^{-1})	1.09	0.72	0.91	0.50
PS (nm)	21 (21) [a]	13 (broad) [b]	14 (20) [a]	13 (broad) [b]

[a] *From number-based, desorption BJH curve. In parentheses from adsorption curve.* [b] *Average from volume-based BJH curves (adsorption and desorption).*

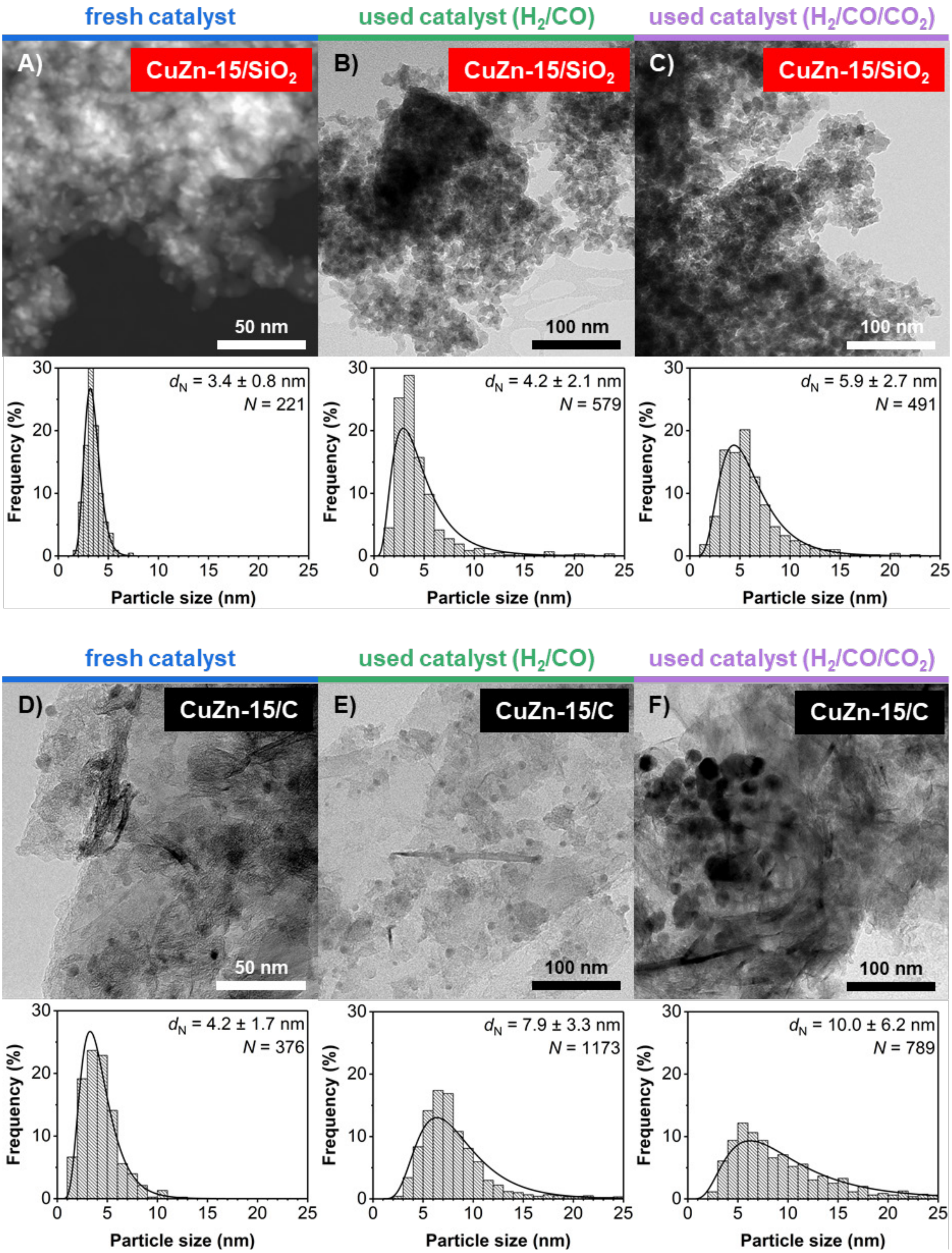

Figure S2 *Representative BF-TEM images with particle size distributions (PSDs) of the (**A**-**C**) CuZn-15/SiO_2 and (**D**-**F**) CuZn-15/C catalysts, in the fresh state and after 100 (or 150) h of catalysis in an H_2/CO or H_2/CO/CO_2 feed. Frame A was obtained from HAADF-STEM. Only the relevant part of the lognormal distribution (>1% of maximum) was considered for the calculation of the average particle sizes. Catalysis conditions were equal to figure S13.*

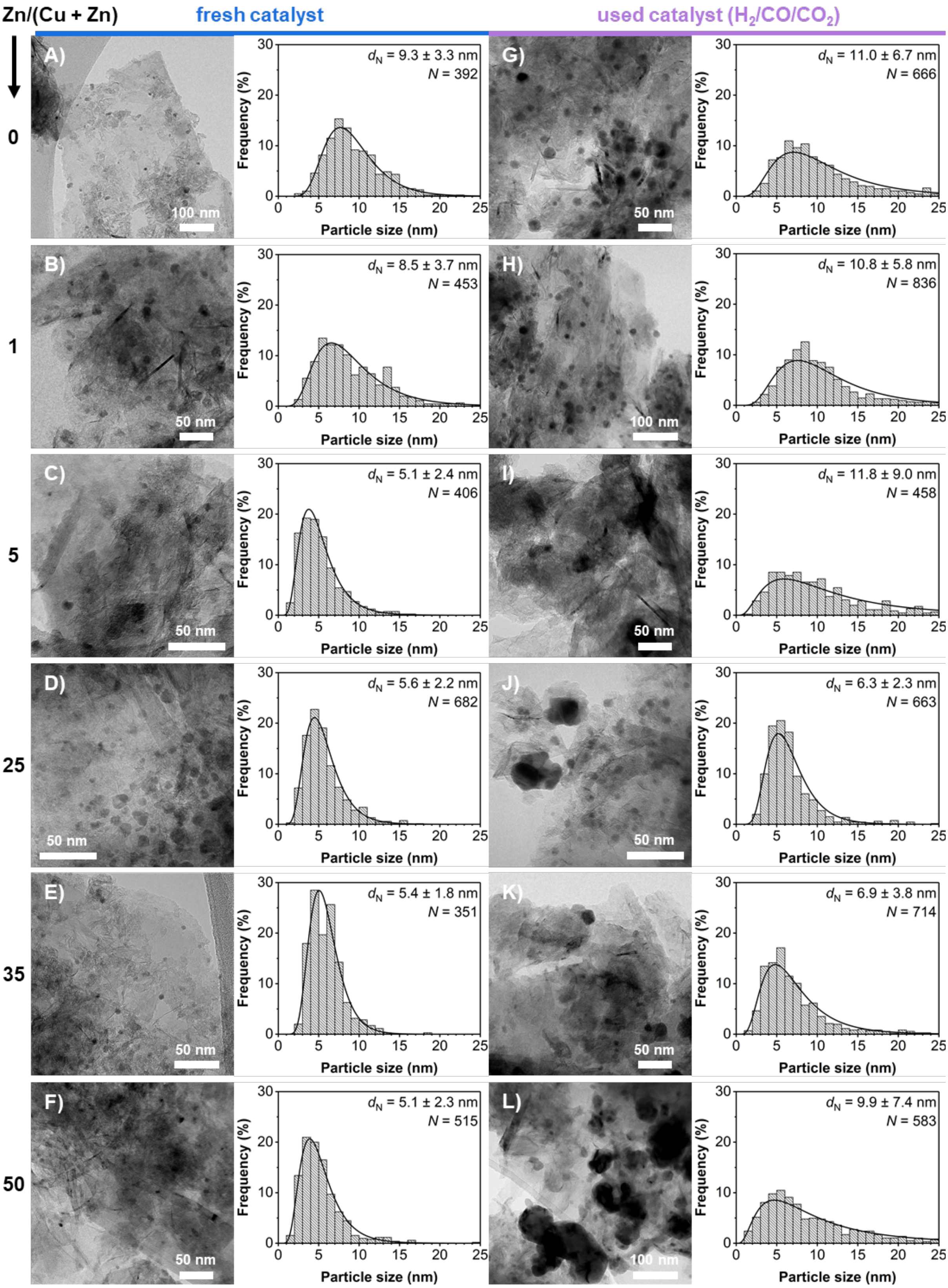

Figure S3 *Representative TEM images with PSDs of CuZnO$_x$/C catalysts (**A-F**) in the fresh state and (**G-L**) after 100 h catalysis in an $H_2/CO/CO_2$ feed (after 150 h for Cu/C, CuZn-1/C and CuZn-25/C catalysts). Only the relevant part of the lognormal distribution (>1% of maximum) was considered for the calculation of the average particle sizes. Catalysis conditions were equal to figure S13. CuZn-15/C is depicted in figure S2.*

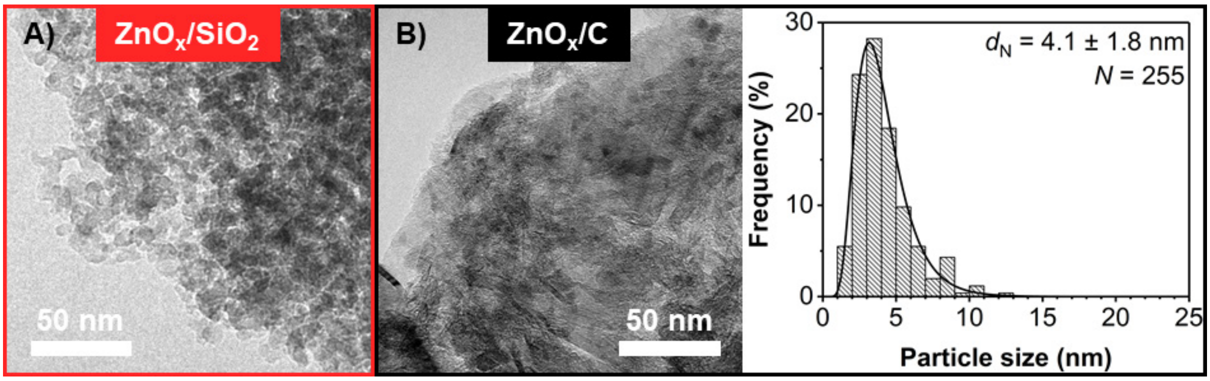

Figure S4 *Representative BF-TEM images of the fresh (**A**) ZnO_x/SiO_2 and (**B**) ZnO_x/C (with corresponding particle size distribution) catalysts. We were unable to define a particle size distribution for the ZnO_x/SiO_2 catalyst with high certainty, as the silica morphology probably resembled the ZnO_x particle shape with limited phase contrast.*

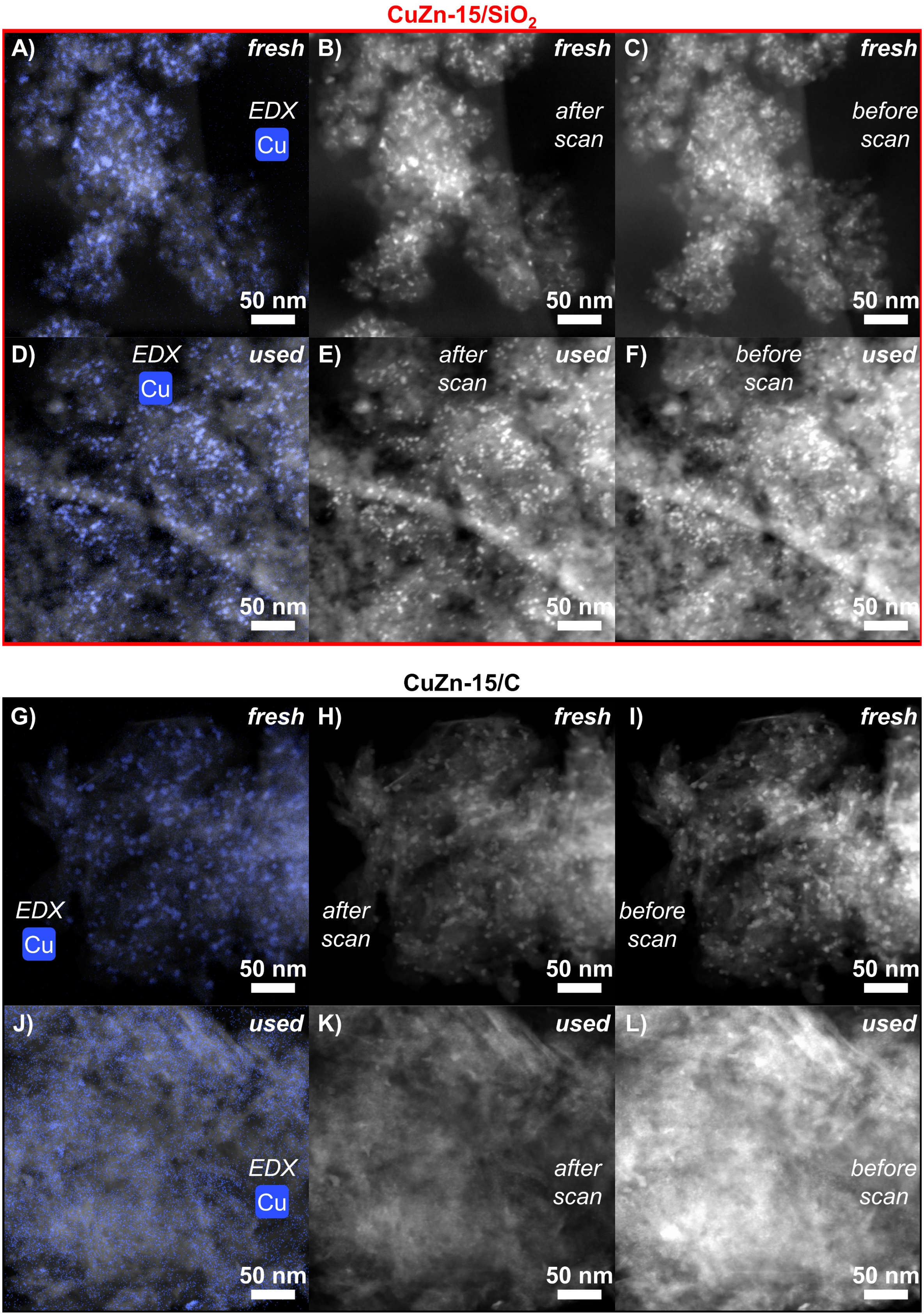

Figure S5 *Zoomed-out HAADF-STEM images based on figure 1 for the (**A**-**F**) CuZn-15/SiO2 and (**G**-**L**) CuZn-15/C catalysts. Frames A, D, G, and J have an blue Cu color mapping based on EDX. The HAADF-STEM images before and after the EDX map did not significantly change the location and size of the supported nanoparticles.*

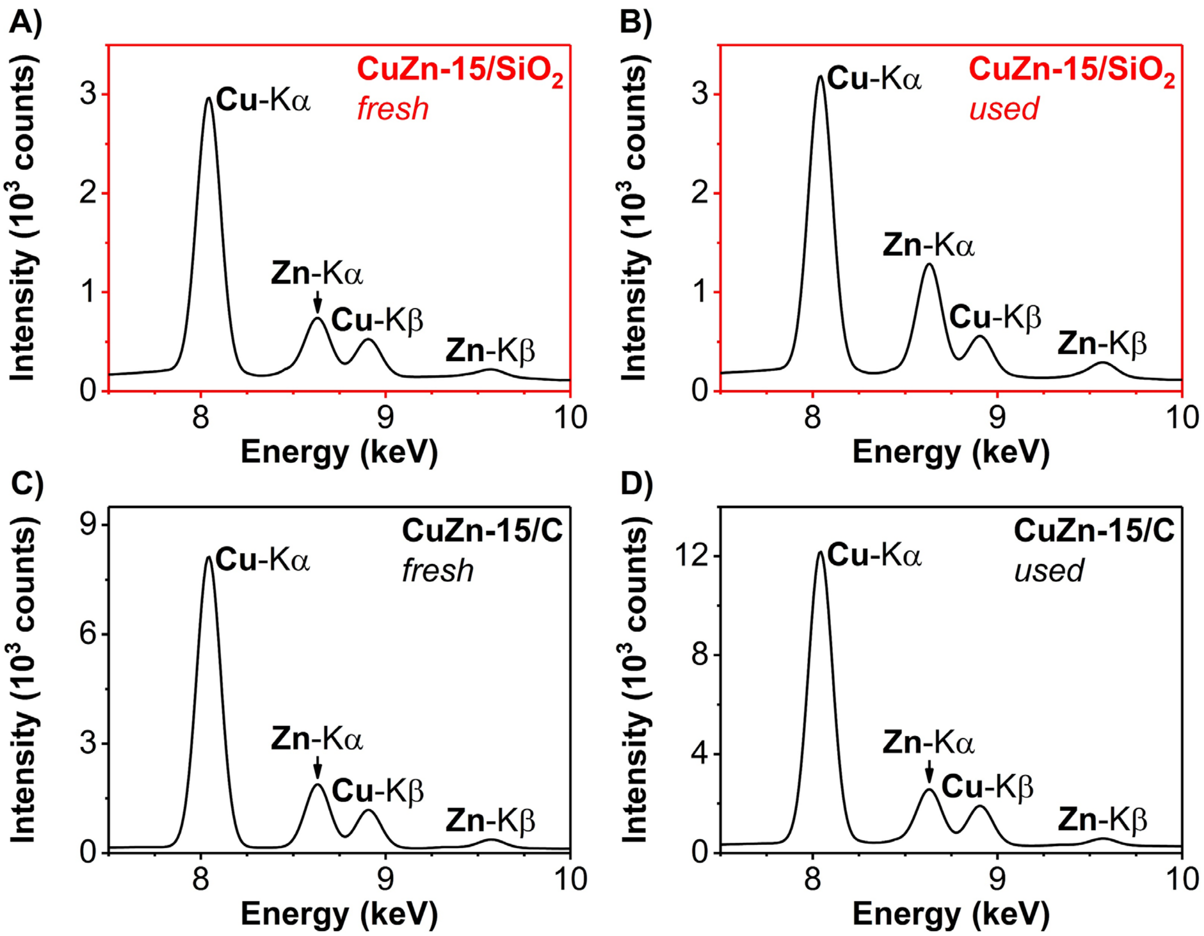

Figure S6 *EDX spectra of (**A**) fresh and (**B**) used CuZn-15/SiO$_2$ catalyst and (**C**) fresh and (**D**) used CuZn-15/C catalyst. These spectra correspond to the EDX maps in frames B, C, E, and F of figure 1 in the main text, respectively. The used catalysts were after 150 and 100 h of catalysis in an H$_2$/CO/CO$_2$ feed, respectively. The (qualitative) Cu/Zn ratio did not change significantly before and after catalysis, suggesting the absence of severe Cu and/or Zn agglomeration.*

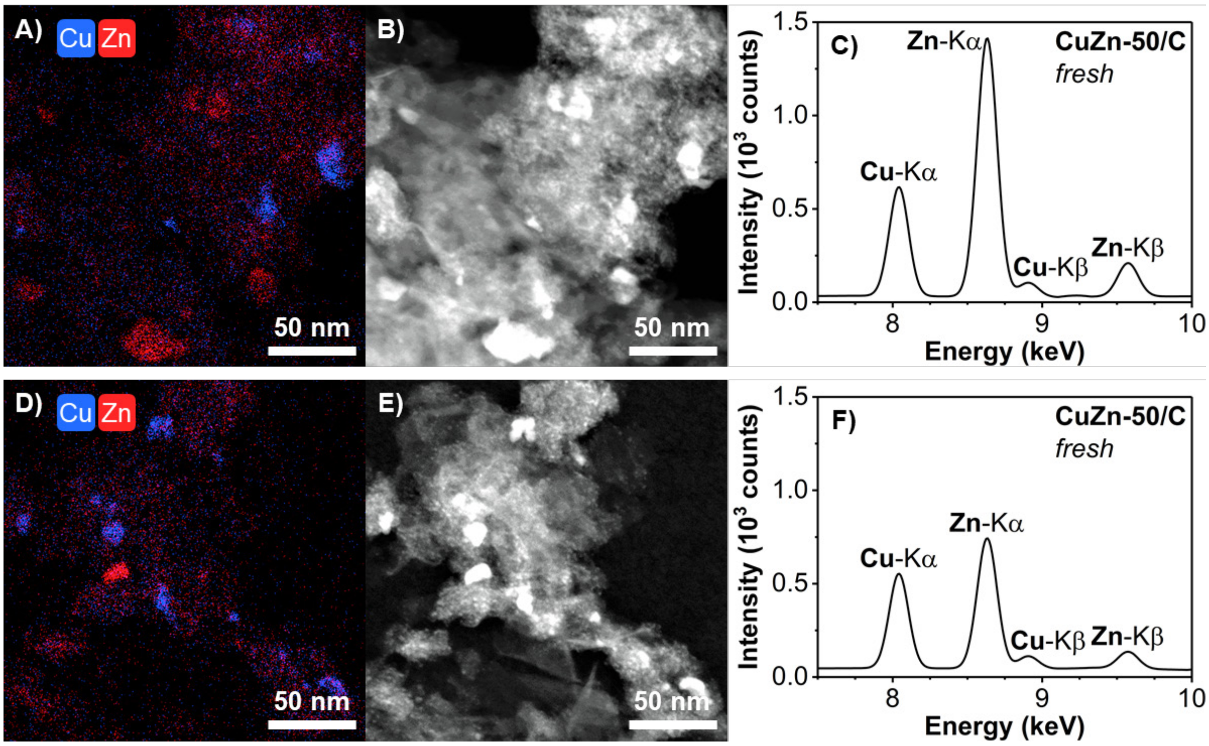

Figure S7 *Electron microscopy analysis of the fresh CuZn-50/C catalyst at two different locations: (**A**-**C**) and (**D**-**F**). (**A**,**D**) EDX maps, (**B**,**E**) HAADF-STEM images, and (**C**,**F**) corresponding EDX spectra. The fresh CuZn-50/C catalyst had several 10+ nm-sized ZnO_x particles due to the high total metal loading. The Zn species were at these high loadings less uniformly distributed over the support surfaces.*

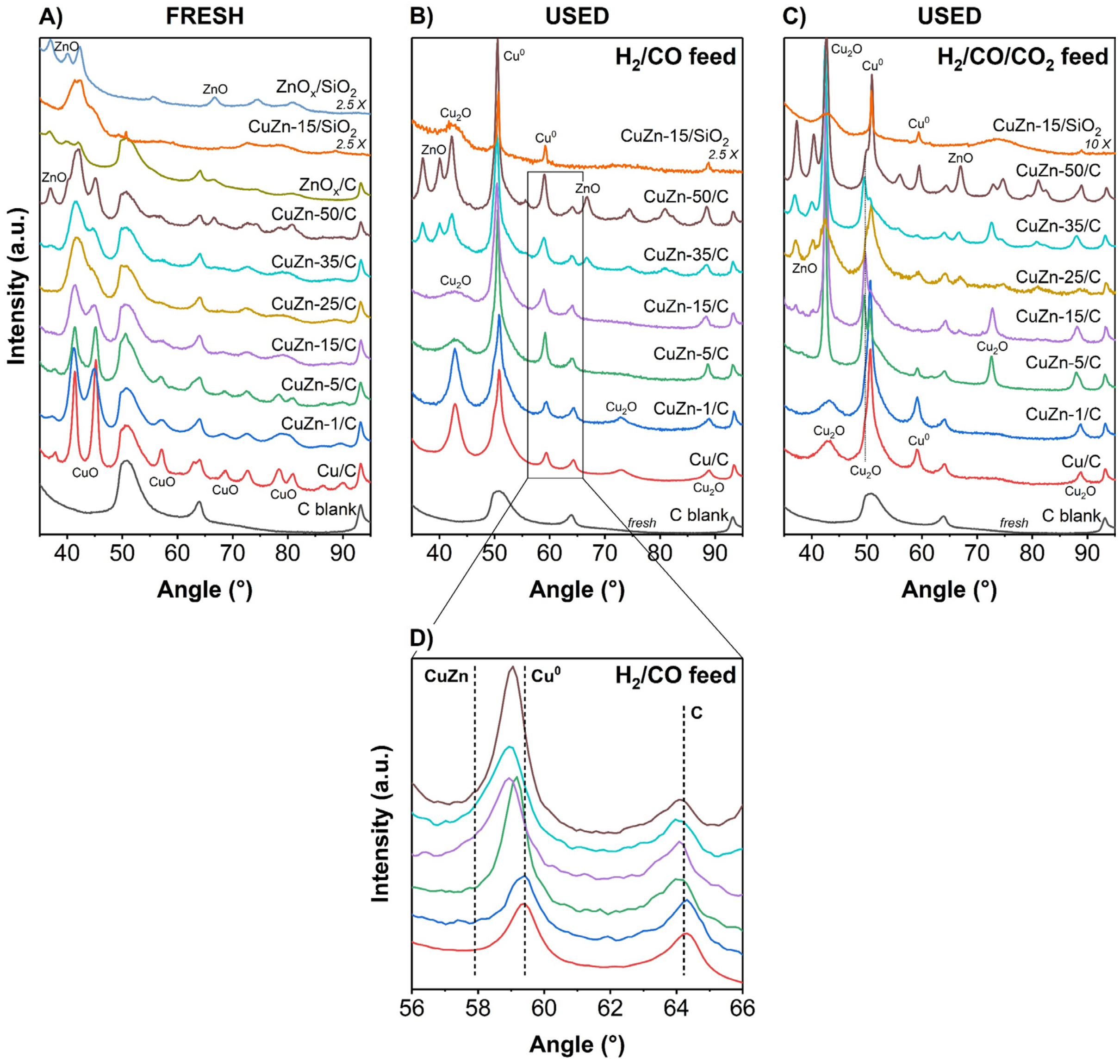

Figure S8 Ex situ *XRD patterns of (**A**) fresh catalysts and (**B**-**C**) used catalysts after 100 h of catalysis in an H_2/CO and $H_2/CO/CO_2$ feed, respectively. (**D**) Zoomed section of frame B (the diffractograms are smoothed for visualization purposes). All diffractograms are vertically offset for clarity. The used Cu/C, CuZn-1/C, and CuZn-25/C catalysts in frame C were obtained after 150 h of catalysis. The used catalysts were measured without any pretreatment and were exposed to ambient conditions. Where the fresh catalysts only contained crystallized CuO and (at high loadings) ZnO, the used catalysts mainly contained highly crystallized Cu^0 and Cu_2O. The XRD patterns indicate that after catalysis in an $H_2/CO/CO_2$ feed the CuO_x and ZnO species crystallized more (frame C) than in an H_2/CO feed (frame B). Also, the small peak shift of the diffraction line from 59.4° to 58.9° 2θ suggests CuZn alloy formation (frame D).*

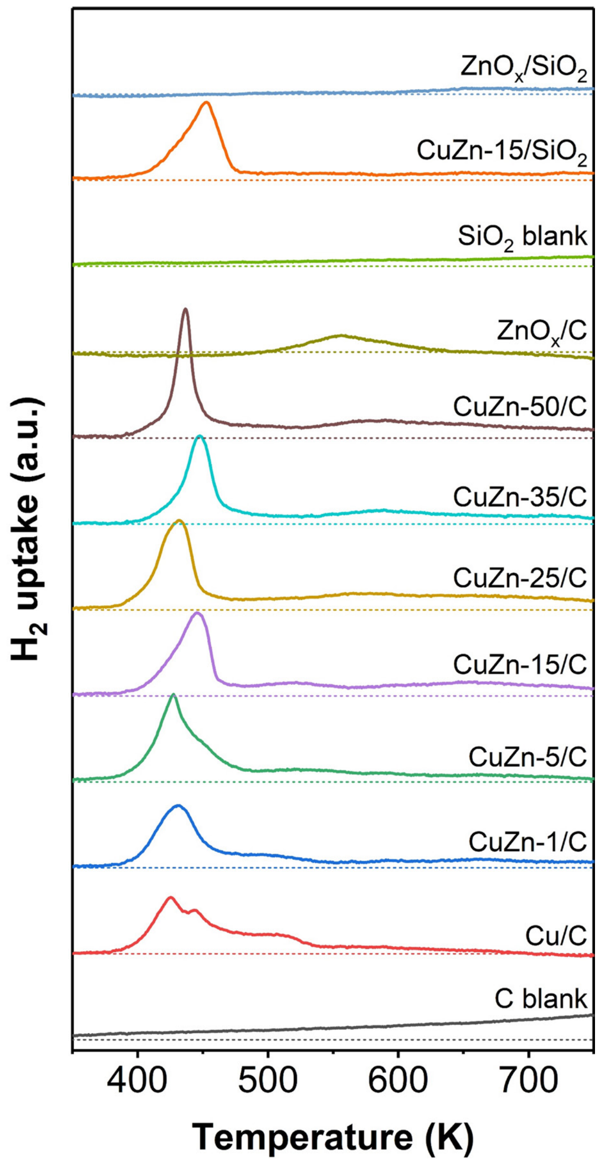

Figure S9 *H_2 reduction profiles of fresh catalysts. The baseline-corrected profiles are vertically offset for clarity and normalized by the carbon or silica support amount. 'C blank' and 'SiO_2 blank' are the H_2 reduction profiles of the corresponding support, impregnated with 0.1 M HNO_3 and activated in the same way as the catalysts. These blank profiles show the effect of gas buoyancy. Conditions: dried at 393 K in Ar, cooled down to RT, reduction with 5 vol% H_2/Ar, ramp of 2 K min^{-1}, 1 L min^{-1} g^{-1} (for CuZn-25/C, ZnO_x/C and ZnO_x/SiO_2: 0.8 L min^{-1} g^{-1}).*

Table S2 *Number- and surface-averaged Cu(Zn)O_x particle sizes of the CuZnO_x/C, CuZn-15/SiO_2, and ZnO_x/SiO_2 catalysts in the fresh and used state (after 100 h of catalysis in an H_2/CO/CO_2 feed) by TEM analysis. Independent of the ZnO_x loading the number-averaged Cu(Zn)O_x particle size was 5.1 nm, while with (almost) no ZnO_x the average size was 8.9 nm. After catalysis, the Cu(Zn)O_x particles grew to an average of 9.5 nm in all catalysts.*

**Zn/(Cu + Zn) molar fraction** *(carbon support)*	**loading (wt%)**		**d_N [CuO] (nm)**		**d_S [CuO] (nm)**	
	Cu	ZnO	fresh	used	fresh	used
0.00	8.1	0	9.3 ± 3.3	11.0 ± 6.7 [a]	9.9 ± 3.3	12.9 ± 7.0 [a]
0.01	8.4	0.1	8.5 ± 3.7	10.8 ± 5.8 [a]	9.3 ± 3.8	12.2 ± 6.0 [a]
0.05	8.4	0.6	5.1 ± 2.4	11.8 ± 9.0	5.7 ± 2.5	14.8 ± 9.5
0.15	8.1	1.8	4.2 ± 1.7	10.0 ± 6.2	4.6 ± 1.8	11.7 ± 6.4
0.25	7.9	3.4	5.6 ± 2.2	6.3 ± 2.3 [a]	6.0 ± 2.2	6.7 ± 2.3 [a]
0.35	7.7	5.3	5.4 ± 1.8	6.9 ± 3.8	5.7 ± 1.8	7.9 ± 3.9
0.50	7.4	9.5	5.1 ± 2.3	9.9 ± 7.4	5.6 ± 2.4	12.4 ± 7.8
1.00	0	9.9	4.1 ± 1.8	–	4.5 ± 1.8	–
CuZn-15/SiO_2	8.0	1.8	3.4 ± 0.8 [b]	5.9 ± 2.7 [a]	3.5 ± 0.8 [b]	6.5 ± 2.8 [a]
ZnO_x/SiO_2	0	10.0	7.7 [c]	–	7.7 [c]	–

[a] *After 150 h of catalysis.* [b] *Obtained from HAADF-STEM images.* [c] *ZnO crystallite size obtained from XRD.*

Note on the catalyst synthesis. The reported elemental composition of the catalysts was based on the nominal metal loading using the mass of the metal precursor solution for the impregnation of the graphitic carbon to incipient wetness. First, the density of each prepared impregnation solution with varying Cu/Zn contents was determined (*ca.* 1.2-1.5 g mL^{-1}). Next, the needed mass of the impregnation solution was calculated, using additional nitrogen physisorption experiments (see Experimental section in the main text), according to the following equation: $m_{imp} = m_{sup} \cdot \rho \cdot \alpha \cdot PV$, in which m_{imp} and m_{sup} are the mass of the impregnation solution and support, respectively, ρ is the density of the impregnation solution, α is the factor of pore filling (*i.e.* 0.95), and PV is the total pore volume. The elemental composition was validated for selected catalysts by additional inductively coupled plasma optical emission spectroscopy (ICP-OES) measurements by Mikrolab Kolbe on a Spectro Arcos apparatus (table S3).

Table S3 *Comparison between the nominal weight loading and ICP-OES results of selected catalysts.*

**sample**	**nominal loading**			**ICP-OES**			
	Cu (wt%)	Zn (wt%)	Zn/(Cu + Zn) molar fraction	Cu (wt%)	Zn (wt%)	dried (wt%) [a]	Zn/(Cu + Zn) molar fraction
CuZn-5/C	8.4	0.4	0.050	8.5	0.4	5.8	0.046
CuZn-15/C	8.1	1.4	0.147	7.9	1.3	4.9	0.142
CuZn-15/SiO_2	8.1	1.5	0.151	7.4	1.4	3.3	0.154
ZnO_x/C	–	8.1	1.000	–	8.0	4.6	1.000

[a] *Weight loss after drying under dynamic vacuum at 423 K for 2 h.*

Section S2: Catalytic activity

Table S4 summarizes the TOF values and accompanying catalyst parameters for the CuZn-15/SiO_2 and CuZn-15/C catalysts under relevant methanol synthesis conditions. The TOF values for the CuZn-15/C catalyst were a few times higher (1.6-5.2 times) than for the catalyst supported on silica, irrespective of the presence of CO_2 in the feed. The activity difference agrees with previous work on low-pressure methanol synthesis using silica and activated carbon supports.[2] Note that the smaller Cu(Zn)O_x particle sizes in the used CuZn-15/SiO_2 catalyst (3.5-8 nm) might partially contributed to an overall lower TOF compared to the used CuZn-15/C catalyst (7-15 nm).[3] The slightly lower conversion

Table S4 *Catalytic performance of the CuZn-15/SiO_2 and CuZn-15/C catalysts, with or without CO_2 in the feed. Errors represent the standard deviation between 2 or 4 separate measurements unless stated otherwise. Conditions: 533 K, 40 bar(g), H_2/CO/He = 60/30/10 vol% or H_2/CO/CO_2/He = 60/27/3/10 vol%.*

**catalyst + initial d_S [CuO]**	**CO_2 in feed**	**flow (mL min^{-1} g_{cat}^{-1})**	**CO (+ CO_2) conv. (%)**		**TOF (10^{-3} s^{-1}) [a]**		**final d_S [CuO] (nm)**
			50 h	100 h	initial [b]	final [c]	
CuZn-15/SiO_2 (3.5 ± 0.8 nm)	no	21.1	12.2	10.4	3.0 ± 0.7	1.6 ± 0.7	4.6 ± 2.1
	yes	63.8	5.8	5.0	2.6 ± 0.6	3.0 ± 1.3 [d]	6.5 ± 2.8 [d]
CuZn-15/C (4.6 ± 1.8 nm)	no	35.2 ± 0.1	9.1 ± 0.4	8.2 ± 0.3	4.9 ± 1.9	3.9 ± 1.5	8.6 ± 3.3
	yes	87.5 ± 0.2	11.6 ± 0.3	9.6 ± 0.6	11.2 ± 4.4	15.3 ± 8.4	11.7 ± 6.4

[a] *Error reflects the width in the CuO particle size distribution.* [b] *Calculated from the average CuO size in the fresh catalyst and activity at t = 0 h.* [c] *Calculated from the average CuO size in the used catalyst and activity at t = 100 h.* [d] *After 150 h of catalysis.*

(5-6%) of the CuZn-15/SiO_2 catalyst in an H_2/CO/CO_2 feed probably did not significantly influence the TOF values (figure S11), as discussed below in more detail.

Figure S10 presents the CO (+ CO_2) conversion (frame A) and the Cu-normalized total activity (frame B) as a function of time for the CuZn-15/SiO_2 and CuZn-15/C catalysts (see figure 2 in the main text for the methanol formation rate). Between 50 and 100 h on stream the conversion level (frame A) of the CuZn-15/SiO_2 catalyst in an H_2/CO feed and the CuZn-15/C catalyst in both feeds were similar (*ca.* 10%).

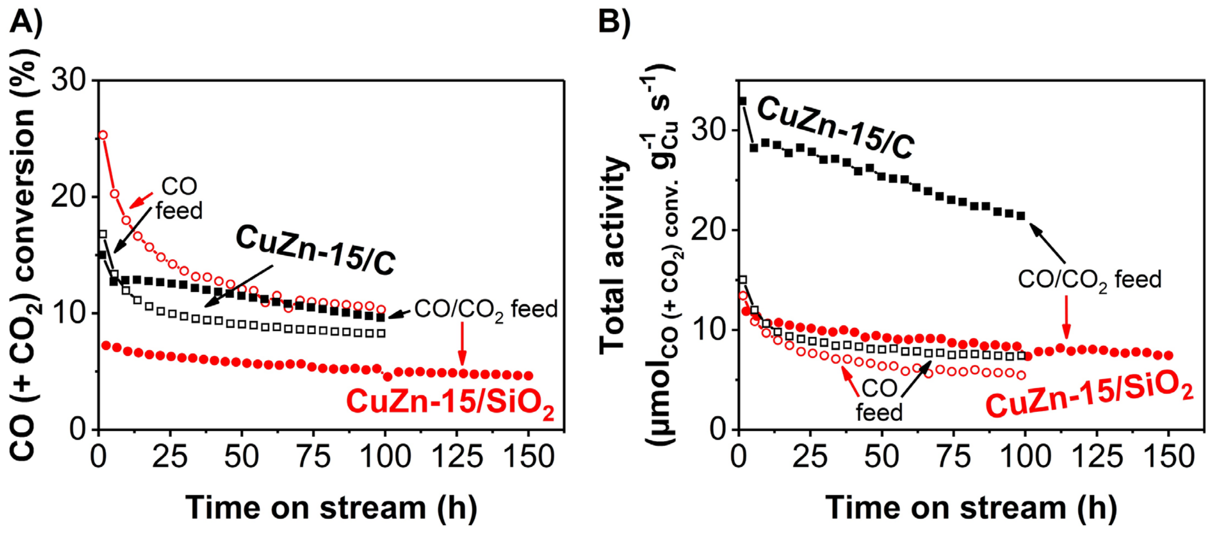

Figure S10 *(**A**) CO (+ CO_2) conversion and (**B**) Cu-normalized total activity of the CuZn-15/SiO_2 catalysts (**red** circles) and CuZn-15/C (**black** squares) in a CO_2-free (open symbols) or -enriched (filled symbols) syngas feed. The data points of the CuZn-15/C catalyst in H_2/CO and H_2/CO/CO_2 are averaged over 4 and 2 separate runs, respectively. Conditions: 533 K, 40 bar(g), H_2/CO/He = 60/30/10 vol% or H_2/CO/CO_2/He = 60/27/3/10 vol%.*

Figure S11 (frame A) shows the CO + CO_2 conversion levels of the CuZn-15/C catalyst as a function of time for various catalyst loadings and SiC-to-catalyst ratios. As expected, the conversion increased by a factor of 2.1 when the catalyst amount in the reactor was doubled. Yet, with a halved catalyst loading the conversion level decreased by a factor of 2.6, indicating that the activity slightly deviated from the linear expectation at low catalyst loadings (*i.e.* high gas hourly space velocities). By increasing the SiC-to-catalyst ratio the conversion lowered by 1.2% but simultaneously doubled the error by SiC dilution[4] from 1.4 to 3.1%. Hence, significant heat transfer limitations were excluded in our experiments.

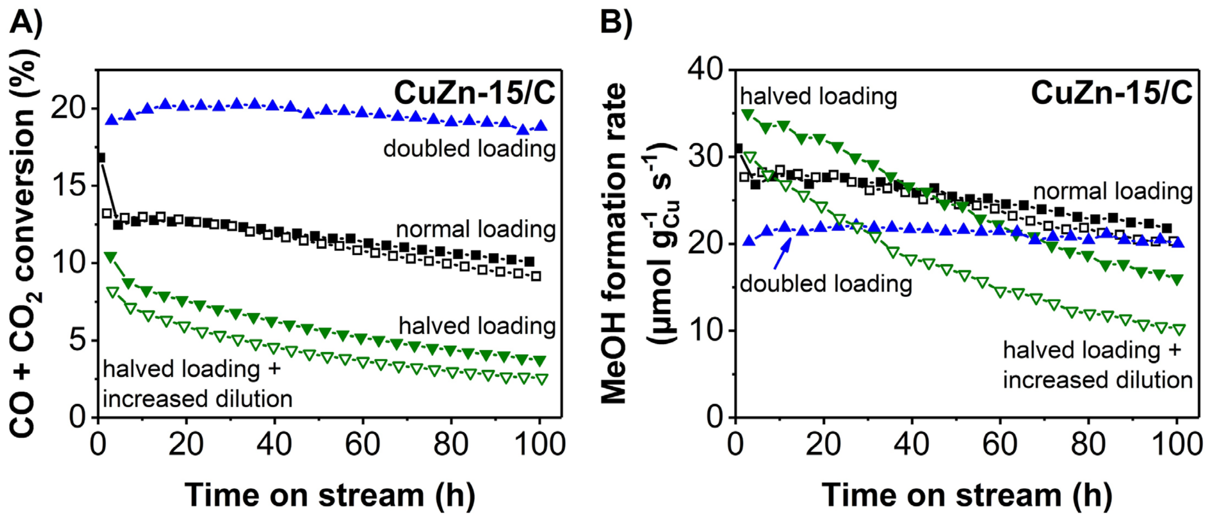

Figure S11 *(**A**) CO + CO_2 conversion and (**B**) methanol formation rate of the CuZn-15/C catalyst at varying GSHVs. Established by using various catalyst loadings under an identical CO_2-enriched syngas flow. The duplicated "normal loading" catalyst runs represent the data presented in the main text. The GHSV is 5,400, 2,700, and 1,400 h^{-1} for halved, normal, and doubled loading, respectively. Conditions: 533 K, 40 bar(g), H_2/CO/CO_2/He = 60/27/3/10 vol%.*

Figure S11 (frame B) shows the methanol formation rate of the differently loaded CuZn-15/C catalysts. The methanol production for the half-loaded reactor decreased faster over time compared to the higher reactor loadings, ascribed to the changing methanol selectivity over time. After 100 h on stream the normal and double loaded CuZn-15/C catalysts yielded similar amounts of methanol. Slightly less methanol was formed for the catalyst with a halved loading (16.0 µmol g_{Cu}^{-1} s^{-1}) compared to the catalyst discussed in the main text (20.8 ± 1.0 µmol g_{Cu}^{-1} s^{-1}). Hence, the CO + CO_2 conversion, expressed by the various gas hourly space velocities, did not have a significant influence on the methanol formation rate. In other words, the TOF of the CuZn-15/SiO_2 catalyst in an H_2/CO/CO_2 gas atmosphere (table S4) might only been slightly underestimated.

Figure S12 shows the total specific activity (frame A) and the product yield (frame B) of the ZnO_x/SiO_2 and ZnO_x/C catalysts (see figure 3 in the main text for the CO (+ CO_2) conversion). As the ZnO_x particle size in both catalysts were slightly different (7.7 *vs* 4.5 nm, respectively), the activity (frame A) was normalized to an estimated ZnO surface area, derived from TEM analysis. Frame B shows that only the ZnO_x/C catalyst significantly produced methanol in both gas mixtures. In an H_2/CO feed a small amount of CO_2 was produced for both catalysts, which was also observed in the case of ZnO/Al_2O_3[5] and

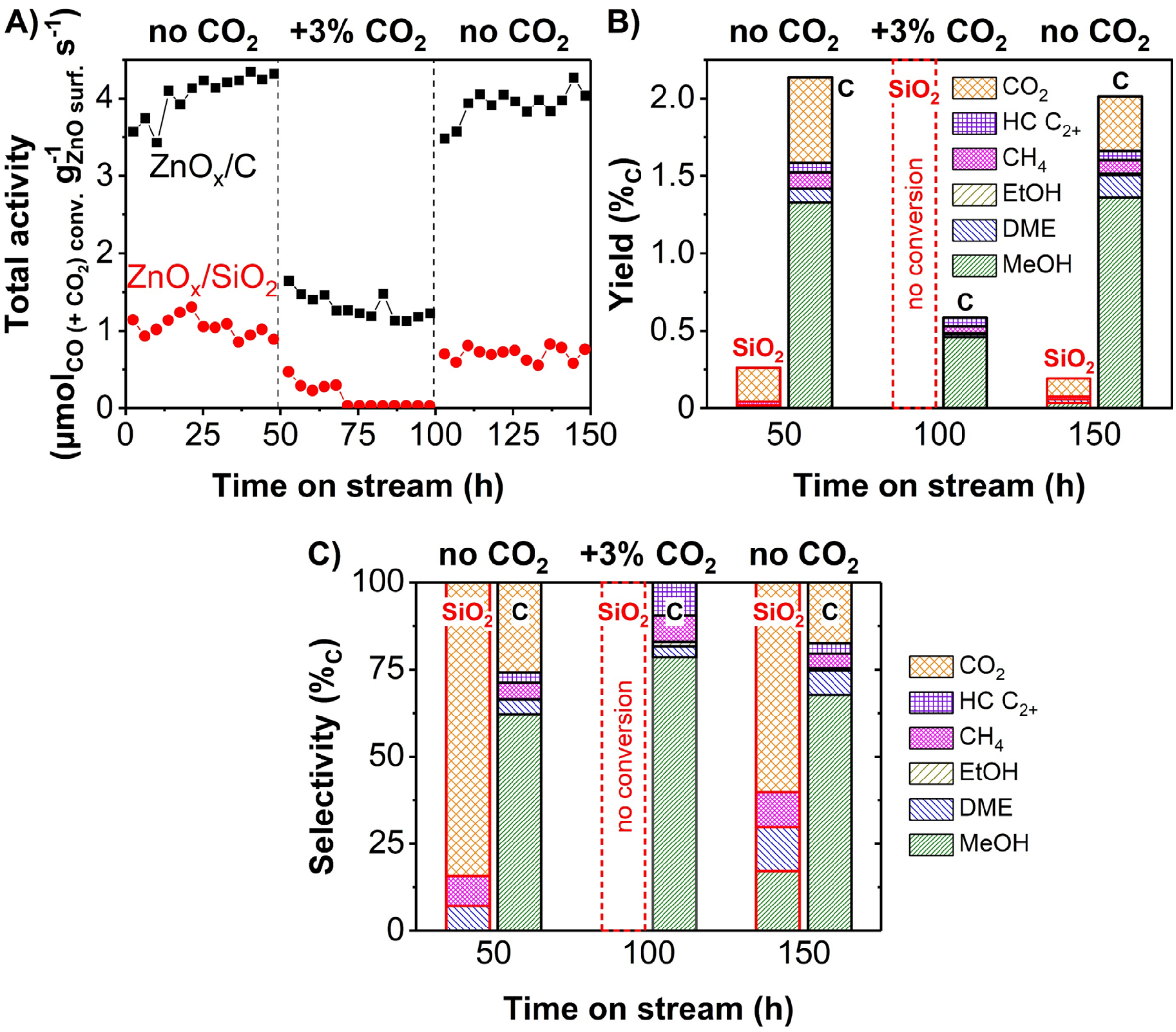

Figure S12 *(**A**) Total activity, normalized by the ZnO surface area, (**B**) carbon-based product yield, and (**C**) carbon-based product selectivity, based of silica- and carbon-supported ZnO_x (both 10 wt%, size of 7.7 and 4.5 nm, respectively) in various syngas compositions. '+3 vol% CO_2' in the total feed corresponds to a $CO_2/(CO + CO_2)$ fraction of 0.10. The ZnO surface area is estimated from the crystallite size (ZnO_x/SiO_2, 7.7 nm) or particle size (ZnO_x/C, 4.5 nm) using $D_{ZnO} \approx 1/d_{ZnO}$, while assuming no ZnO_x particle growth took place. The ZnO_x/C catalyst was more active than the ZnO_x/SiO_2 catalyst, even corrected by the ZnO surface area. Conditions: 533 K, 40 bar(g), $H_2/(CO + CO_2)/He$ = 60/30/10 vol%, 291 mL min^{-1} g_{ZnO}^{-1}.*

Cu(Zn)O_x/C.[1] The oxygen source for the CO_2 might originate from the minor hydrocarbon and ethanol production *via* 2*x*CO + (*x*+1)H_2 → *x*CO_2 + C_xH_{2x+2} and from the minor dimethyl ether (DME) production coupled with the water-gas shift (WGS) reaction *via* $2CH_3OH + CO \rightarrow (CH_3)_2O + CO_2 + H_2$. However, the sum of the DME, ethanol, and hydrocarbon yields was lower than the CO_2 yield after 50 h of catalysis (frame B), showing that an additional oxygen source for the CO_2 production was present,

which most probably originated from ZnO_x or Zn silicates itself.[5] After 150 h of catalysis the CO_2 yield slightly dropped, suggesting that indeed probably a Zn(II) reduction has taken place during high-pressure syngas conversion.

Finally, figure S13 shows the Cu mass-normalized total activity of the $CuZnO_x/C$ catalysts (**black** symbols) and the metal oxide-supported CuZn-15/SiO_2 and commercial Cu/ZnO/Al_2O_3/MgO (com cat) catalysts (**red** circles). The catalytic activities are presented in a CO_2-free as well as in a CO_2-enriched syngas feed. Similarly, figure S14 depicts the TOF values in both gas atmospheres (frame A) and the methanol-specific ones (TOF_{MeOH}) at the start and end of catalysis in an $H_2/CO/CO_2$ feed (frame B).

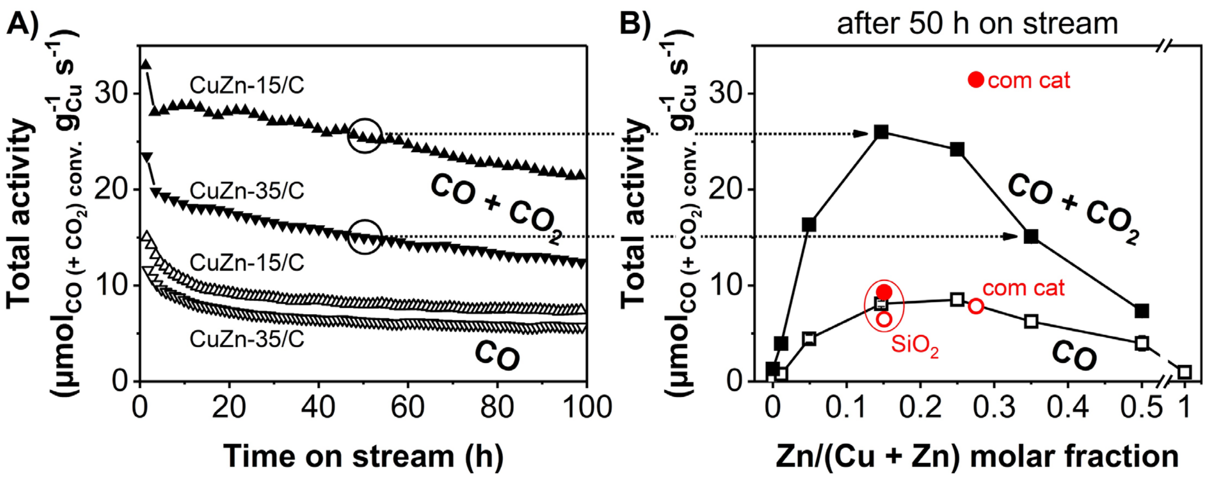

Figure S13 *Cu-normalized total activity of $CuZnO_x/C$ catalysts (**black** symbols) in an H_2/CO or $H_2/CO/CO_2$ feed as a function of (**A**) time and (**B**) ZnO_x loading after 50 h on stream. All data in frame A are the average over 2 or 4 separate measurements. Cu loading = 8.0 ± 0.4 wt%. The 9.9 wt% ZnO_x/C catalyst (X_{CO} = 2.1%) in frame B is normalized per gram of ZnO. 'Com cat' = commercial Cu/ZnO/Al_2O_3/MgO catalyst. 'SiO_2' = CuZn-15/SiO_2 catalyst. Conditions: 533 K, 40 bar(g), $H_2/CO/He$ = 60/30/10 vol% or $H_2/CO/CO_2/He$ = 60/27/3/10 vol%, $X_{CO\ (+\ CO2)}$ = 6-12% after 50 h on stream.*

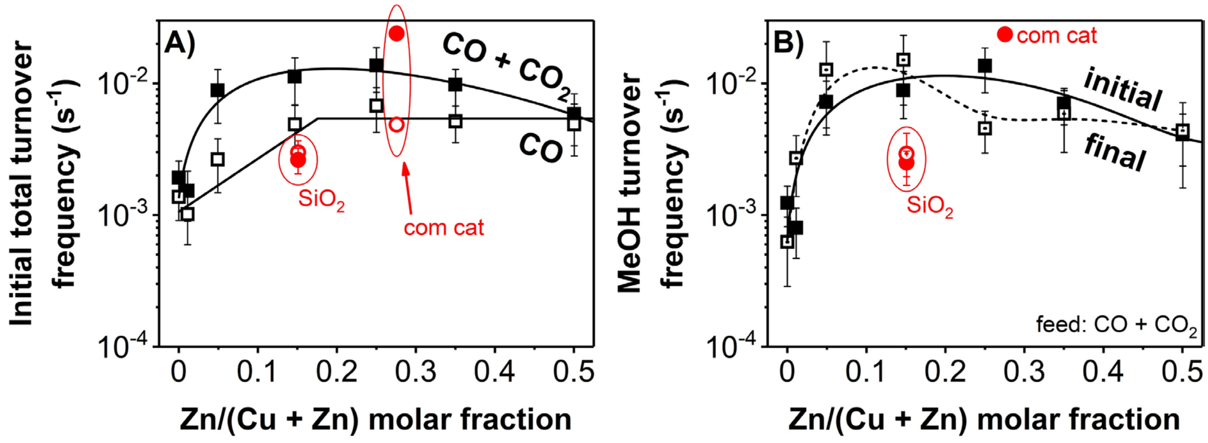

***Figure S14** (**A**) Initial total turnover frequency (TOF). Especially in the H_2/CO feed an increase is observed with respect to the initial methanol turnover frequency (TOF_{MeOH}) in figure 4, due to initial structural changes. The trends are similar. (**B**) TOF_{MeOH} in initial and final state (after 100-150 h of catalysis in H_2/CO/CO_2). There is little change.*

Section S3: Catalyst stability

Cu particle growth[6,7] but also loss of ZnO_x promoting effects[8,9] are well-known modes of deactivation in Cu-based methanol synthesis catalysts. Electron microscopy analysis in figure S2 (see also table S4) shows that the CuO particles in the CuZn-15/SiO_2 catalyst grew less upon methanol synthesis (factor 1.2±0.4 – 1.6±0.4) than in the CuZn-15/C catalyst (factor 1.7±0.5 – 2.1±0.6), irrespective of the presence of CO_2 in the feed. This demonstrates that the higher interaction between the $CuZnO_x$ particles and the SiO_2 support than with the carbon support retarded the particle growth. Also, the flat morphology of the graphite may probably have enabled the pronounced particle growth. Finally, on both supports the particles grew to a larger extent in CO_2-enriched syngas than while hydrogenating pure CO (factor 1.3±0.5), induced by the higher H_2O partial pressure content in the system.[10,11]

Figure S15 presents the stability for the $CuZnO_x$/C catalyst series, the CuZn-15/SiO_2 catalyst and a commercial Cu/ZnO/Al_2O_3/MgO catalyst in H_2/CO and H_2/CO/CO_2 feeds. The stability is defined as the ratio between the activity after 100 h and after 50 h on stream, thereby omitting any stabilization effects in the first 50 h on stream. The destabilizing effect of CO_2 enrichment of the feed was not limited to a Zn/(Cu + Zn) molar fraction of 0.15, but it was also observed for highly ZnO_x-loaded catalysts. In all cases the used catalysts had larger Cu(Zn)O_x particles (figure S3) and larger Cu^0 crystallites (figure S8) in an H_2/CO/CO_2 feed than after pure CO hydrogenation. The stability of the CuZn-15/SiO_2 catalyst in

a CO_2-enriched syngas feed was slightly higher than expected, probably induced by its lower conversion level of 5.0% instead of 10.4% (table S4) and hence a lower H_2O content in the reactor.

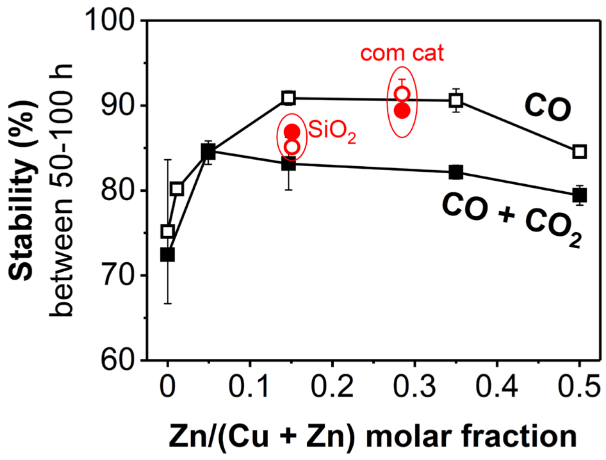

Figure S15 *Stability between 50 and 100 h on stream of CuZnO$_x$/C catalysts (**black** squares), the CuZn-15/SiO_2 catalyst, and a commercial Cu/ZnO/Al_2O_3/MgO catalyst (com cat) (**red** circles) in a CO_2-free or -enriched syngas feed. Error bars represent the standard deviation between two separate measurements. Conditions: 533 K, 40 bar(g), H_2/CO/He = 60/30/10 vol% or H_2/CO/CO_2/He = 60/23/7/10 vol%. After 50 h on stream all catalysts had a CO (+ CO_2) conversion of* ca. *10%, unless stated otherwise in table S4.*

Section S4: X-ray absorption spectroscopy

Figure S16 shows the time-resolved, *in situ* XAS spectra at both the Cu and Zn K-edges for the CuZn-15/SiO_2, CuZn-15/C, and CuZn-5/C catalysts. The Cu oxidation state evolutions by linear combination fitting (LCF) and multivariate analysis (MCR-ALS) are presented in figures S17-S18. In figures S19 and S21 the normalized XANES spectra and first derivatives are shown at all important timestamps during the XAS experiments at the Zn and Cu K-edges, respectively, and in figure S22 the catalysts are compared in a single frame. The estimated Zn oxidation numbers (ONs) are presented in table S5. Figure S20 shows the MCR-ALS analysis at the Zn K-edge. Figures S23-S24 depict the EXAFS-derived *R*-spaces at the same timestamps at again the Zn and Cu K-edges, respectively. The EXAFS fitting results are shown in tables S6-S9 for the references and the CuZn-15/SiO_2, CuZn-15/C, and CuZn-5/C catalysts, respectively. The gas composition in the effluent of the XAS reactor is given in figure S25. Infrared spectra of selected catalysts are shown in figure S26. This supplementary section ends with a description of the XAS data analysis, additional remarks, and the experimental method and XRD analysis (figure S27) of the in-house synthesized macrocrystalline Zn_2SiO_4 and $ZnCO_3$ references.

Figure S17 shows the evolution of the Cu oxidation state during an H_2 treatment as a function of temperature for the CuZn-15/SiO_2 (frame A), CuZn-15/C (frame B), and CuZn-5/C (frame C) catalysts by fitting the data to macrocrystalline references (LCF).The CuO_x species were in all three catalysts completely reduced to Cu^0 around 540 K, as in agreement with the *ex situ* H_2 reduction measurements under similar conditions (figure 5 in the main text). The Cu oxidation state changed at slightly lower temperatures as they were directly detected by X-rays, whereas during *ex situ* H_2 reduction there was an intrinsic, small time delay between the consumed H_2 and its detection downstream. The Cu species in the CuZn-15/SiO_2 catalyst (frame A) were probably slightly reduced at room temperature in an H_2/He atmosphere, possibly caused by partial reduction by the X-rays[12] and/or by mild drying of specific mixed copper silicates, thereby changing the local electronic structure. In both carbon-supported catalysts (frames B-C) the CuO reduced in a two-step process to Cu^0 *via* the formation of Cu^+. The retarded CuO_x reduction above 450 K in the CuZn-15/C catalyst (frame B) might indicate that a fraction of the Cu species was in close contact with ZnO_x species.[13] The Cu oxidation state evolutions are supported by the concentration profiles of extracted components using multivariate analysis (MCR-ALS), as shown in figure S18. Overall, the eigenspectra correspond well to the macrocrystalline Cu references, proving that both LCF and MCR-ALS analyses are appropriate techniques to describe the XAS data. Hence, both analysis techniques show that the support has a large influence on the reducibility of the ZnO_x-promoted Cu species in an H_2 atmosphere.

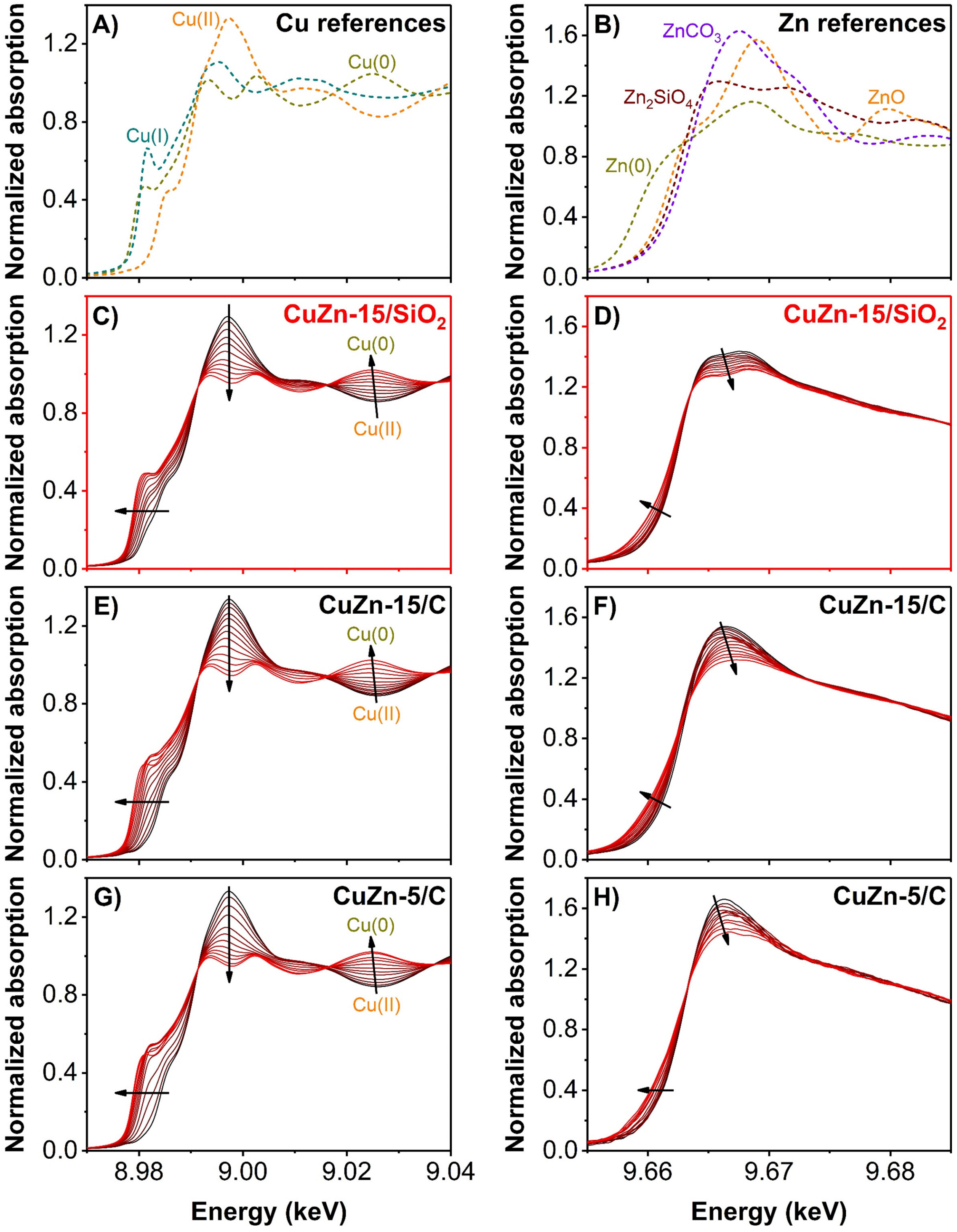

Figure S16 *Time-resolved XANES spectra at the Cu and Zn K-edges of the (**A**-**B**) macrocrystalline references in air at 298 K, and the (**C**-**D**) CuZn-15/SiO*$_2$*, (**E**-**F**) CuZn-15/C and (**G**-**H**) CuZn-5/C catalysts during an H*$_2$ *treatment (solid lines from black to red). Conditions: 20 vol% H*$_2$*/He, from 298 to 541 K (ramp 5 K min*$^{-1}$*), 1 bar.*

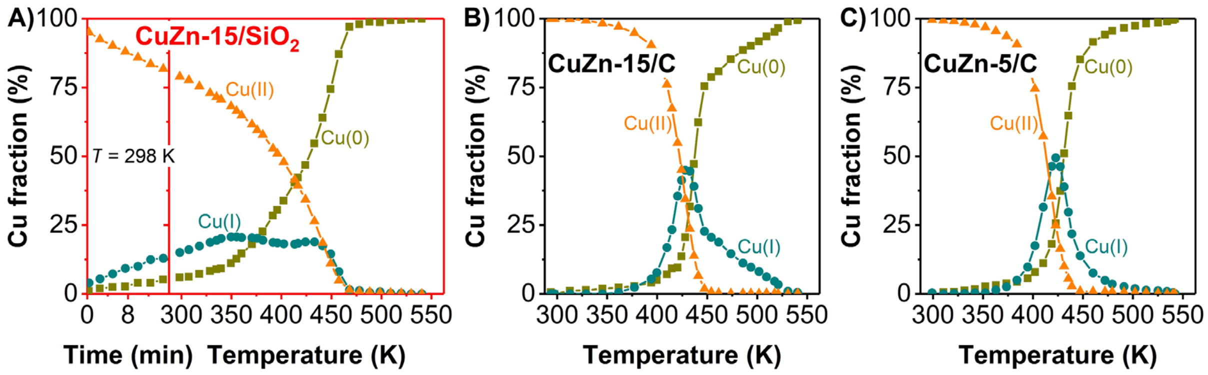

Figure S17 *Cu oxidation state evolutions during an H_2 treatment by linear combination fittings (LCFs) of time-resolved,* in situ *XANES spectra of the (**A**) CuZn-15/SiO_2, (**B**) CuZn-15/C, and (**C**) CuZn-5/C catalysts. Conditions:* ca. *4 L min^{-1} g_{cat}^{-1} flow of 20 vol% H_2/He at 5 K min^{-1} and 1 bar.*

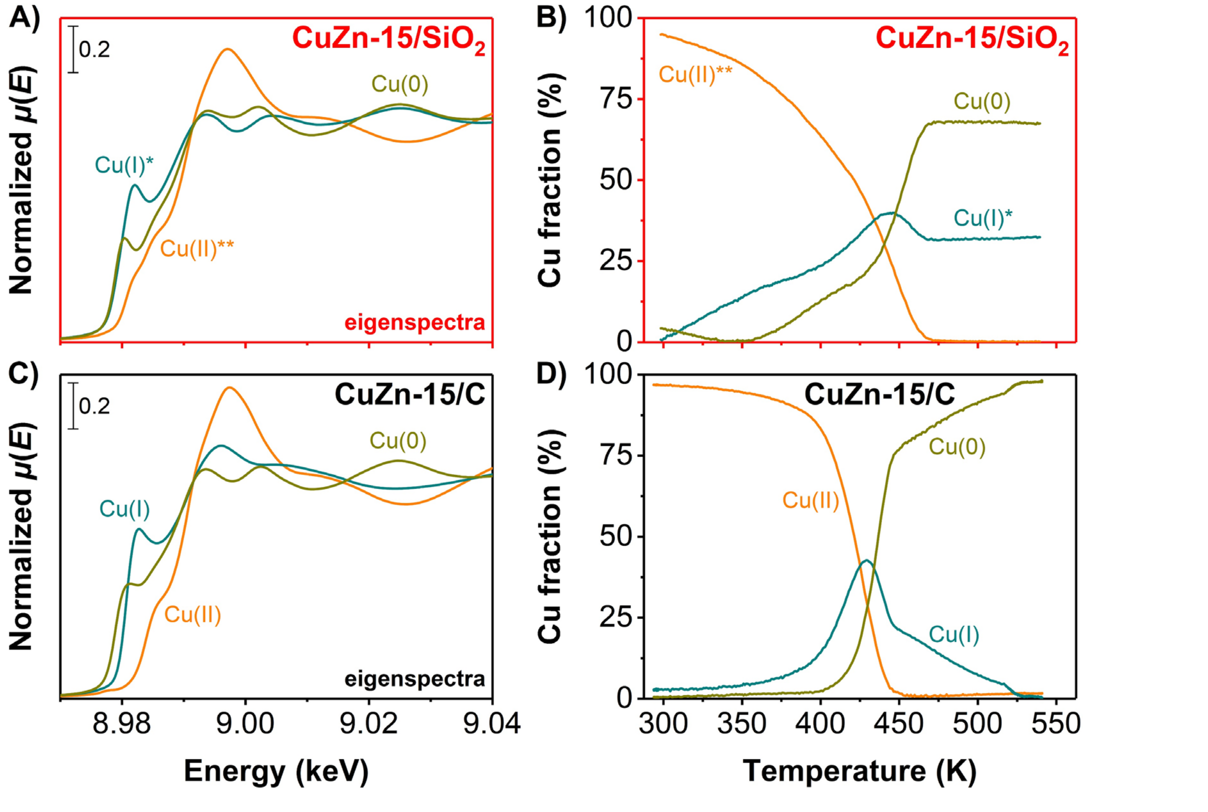

Figure S18 *(**A**,**C**) Extracted eigenspectra and (**B**,**D**) corresponding concentration profiles by multivariate analysis (MCR-ALS) of time-resolved,* in situ *XANES spectra at the Cu K-edge of the (**A**-**B**) CuZn-15/SiO_2 and (**C**-**D**) CuZn-15/C catalysts. The eigenspectra, obtained after 500 iterations, correspond well to the macrocrystalline Cu references (see figure S16 (frame A)). * Eigenspectrum mostly resembles macrocrystalline Cu_2O but also contains features corresponding to metallic Cu. A small amount of Cu_2O in the reduced CuZn-15/SiO_2 catalyst cannot be excluded but supports a stronger interaction of Cu with silica than with carbon. ** Eigenspectrum mostly corresponds to macrocrystalline CuO and describes additional features in the whiteline, which might be ascribed to copper silicate.*

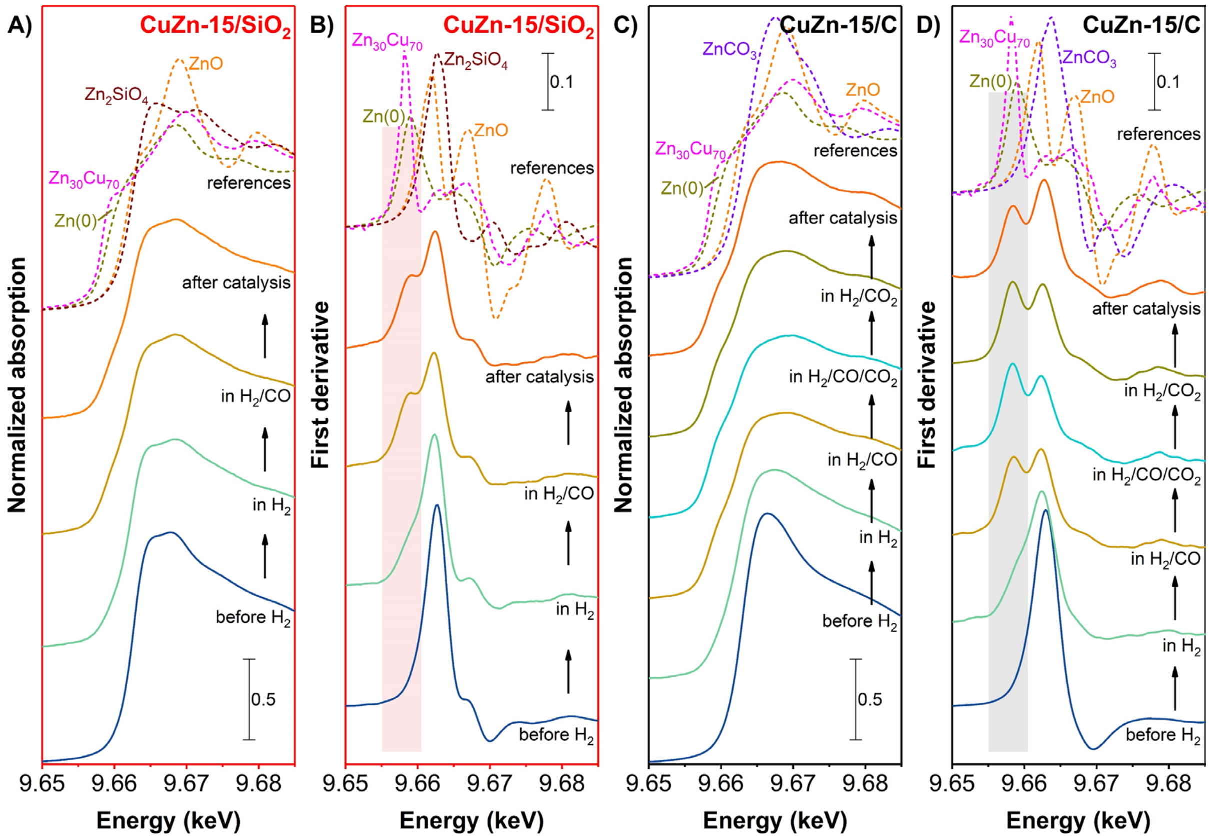

Figure S19 Operando *XANES spectra and their first derivatives at the Zn K-edge of the (**A-B**) CuZn-15/SiO₂ and (**C-D**) CuZn-15/C catalyst (solid lines). Depicted (1) in the initial state at 298 K, (2) in an H_2 atmosphere at 453 K after an H_2 treatment (for conditions, see figure S16), (3-5) during a high-pressure H_2/CO, H_2/CO/CO_2, and H_2/CO_2 exposure at 533 K, respectively, and (6) after catalysis in an H_2/CO or H_2/CO_2 feed at 20 bar and 298 K. States (4) and (5) were only obtained for the CuZn-15/C catalyst. Dashed lines depict macrocrystalline ZnO, Zn_2SiO_4, $ZnCO_3$, $Zn_{30}Cu_{70}$, and Zn foil references at 298 K. Conditions steps (3-5): H_2/CO/He = 60/30/10 vol%, H_2/CO/CO_2/He = 60/27/3/10 vol% and H_2/CO_2/He = 66.5/22.5/10 vol%, respectively, 533 K, 20 bar, 120-160 min per gas feed.*

Table S5 *Estimated contributions of Zn-based species and average Zn oxidation number (ON) during XAS analysis based on LCF. The Zn ON in CuZn-15/C is much lower upon syngas exposure than in the CuZn-15/SiO₂ catalyst.*

**stage during**	**CuZn-15/SiO₂**					**CuZn-15/C**				
**XAS analysis**	Zn(0)	ZnO	Zn_2SiO_4	χ^2	Zn ON	Zn(0)	ZnO	$ZnCO_3$	χ^2	Zn ON
before H_2	0 ± 2	15 ± 4	85 ± 6	0.82	2.00 ± 0.19	4 ± 3	19 ± 5	77 ± 4	1.55	1.92 ± 0.14
in H_2	18 ± 2	9 ± 3	72 ± 5	0.51	1.63 ± 0.16	36 ± 3	31 ± 5	32 ± 4	1.33	1.27 ± 0.13
in H_2/CO	44 ± 2	4 ± 3	52 ± 5	0.53	1.11 ± 0.16	71 ± 3	25 ± 4	4 ± 4	0.98	0.59 ± 0.11
in H_2/CO/CO_2	N.A.	N.A.	N.A.	N.A.	N.A.	78 ± 2	22 ± 4	0 ± 3	0.94	0.45 ± 0.11
in H_2/CO_2	N.A.	N.A.	N.A.	N.A.	N.A.	72 ± 2	24 ± 4	4 ± 3	0.89	0.56 ± 0.11
after catalysis	39 ± 2	6 ± 3	55 ± 5	0.59	1.22 ± 0.16	61 ± 3	25 ± 4	14 ± 4	1.03	0.78 ± 0.12

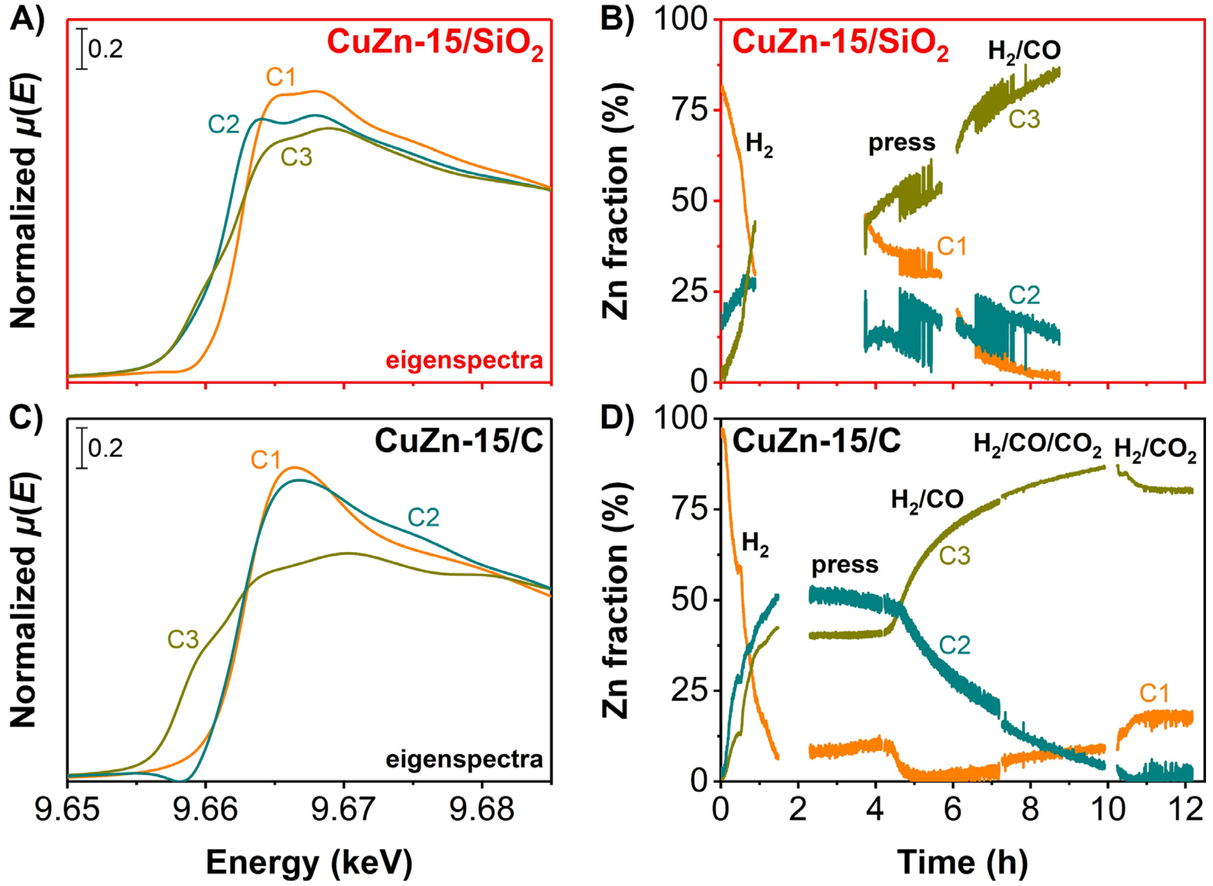

Figure S20 (***A***,***C***) *Extracted eigenspectra and* (***B***,***D***) *corresponding concentration profiles by multivariate analysis (MCR-ALS) of time-resolved, XAS spectra at the Zn K-edge of the* (***A***-***B***) *CuZn-15/SiO*$_2$ *and* (***C***-***D***) *CuZn-15/C catalysts. The concentration profiles are presented at various stages during the experiments (*H_2 *= during an* H_2 *treatment from 298 to 543 K; press = during pressurization to 20 bar in an* H_2*/CO feed at 478 K;* H_2/CO_x *= during catalysis at 533 K and 20 bar). The extracted components do not resemble pure Zn-based compounds (see figure S19 (frame A)) but either represent a relative stable combination of various compounds. Deviations can also be due to differences in the particle size, temperature effects, the relatively weak XAS signal, and the interference with Cu forming metastable phases. The eigenspectrum of component C2 in frame A mostly represents* Zn_2SiO_4 *and its contribution is relatively stable throughout the experiment, indicating the presence of a substantial amount of Zn spectator species in the CuZn-15/SiO*$_2$ *catalyst.*

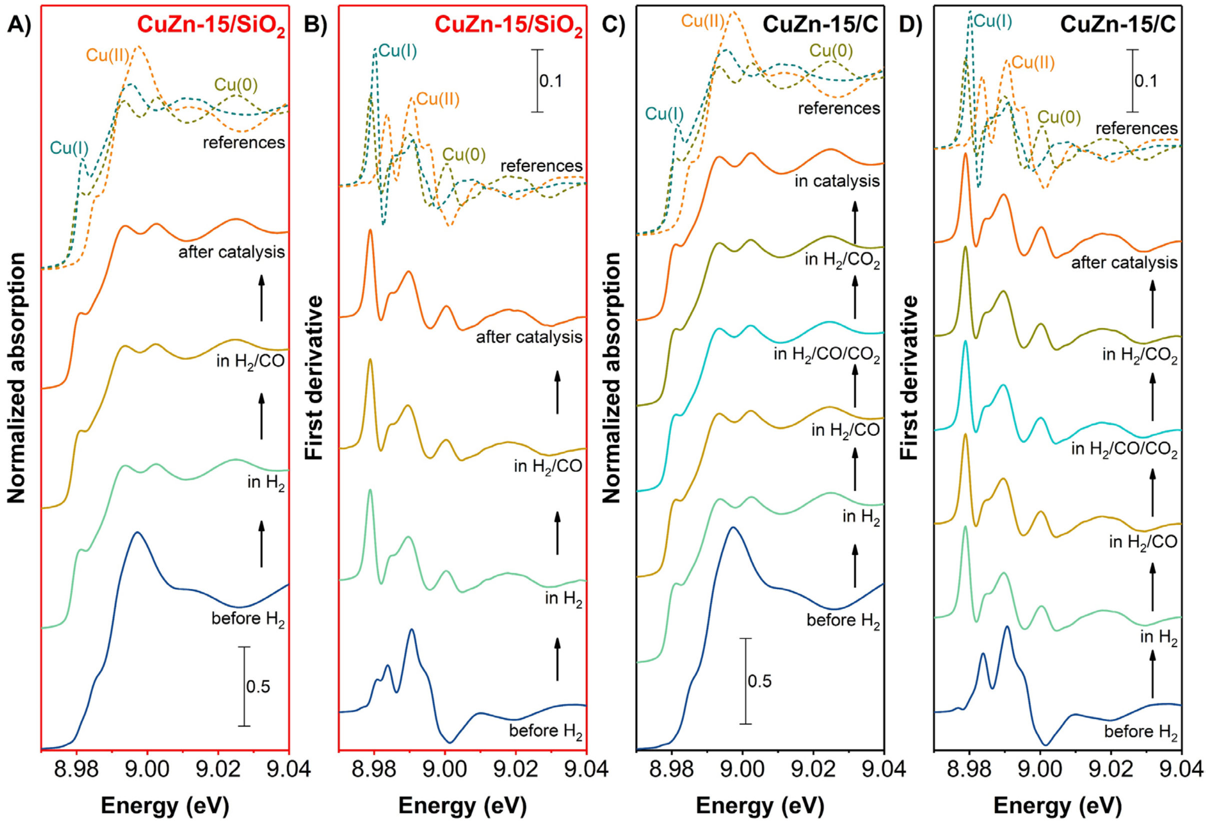

Figure S21 Operando *XANES spectra and their first derivatives at the Cu K-edge of the (**A**-**B**) CuZn-15/SiO2 and (**C**-**D**) CuZn-15/C catalyst (solid lines). Depicted (1) in the initial state at 298 K, (2) in an H2 atmosphere at 453 K after an H2 treatment (for conditions, see figure S16), (3-5) during a high-pressure H2/CO, H2/CO/CO2, and H2/CO2 exposure at 533 K, respectively, and (6) after catalysis in an H2/CO or H2/CO2 feed at 20 bar and 298 K. States (4) and (5) were only obtained for the CuZn-15/C catalyst. Dashed lines depict macrocrystalline CuO, Cu2O, and Cu foil references at 298 K. Conditions steps (3-5): H2/CO/He = 60/30/10 vol%, H2/CO/CO2/He = 60/27/3/10 vol% and H2/CO2/He = 66.5/22.5/10 vol%, respectively, 533 K, 20 bar, 120-160 min per gas feed.*

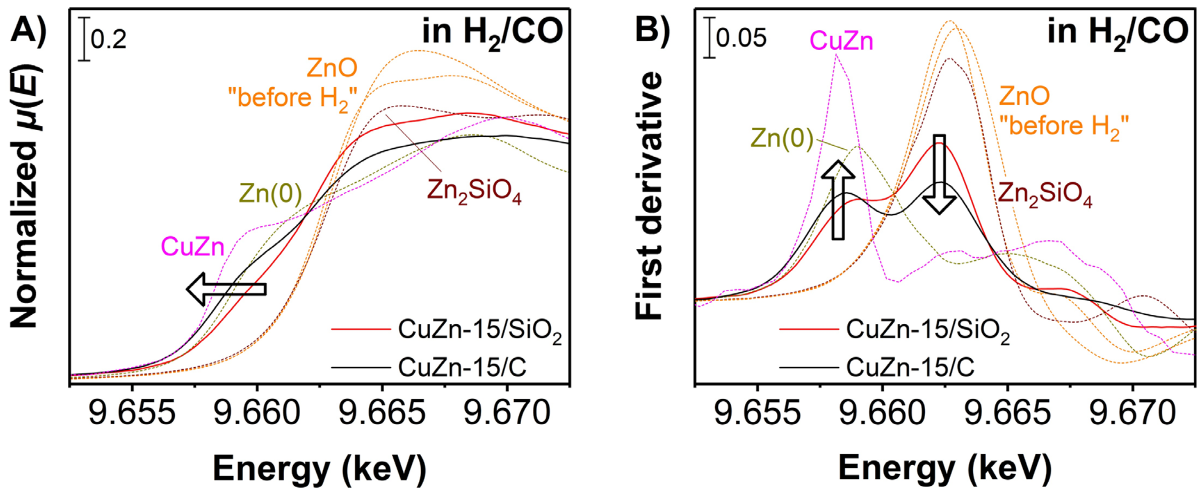

Figure S22 *Condensed version of figure 7 in the main text: (**A**)* operando *XANES spectra and (**B**) their derivatives of the CuZn-15/SiO2 and CuZn-15/C catalysts.*

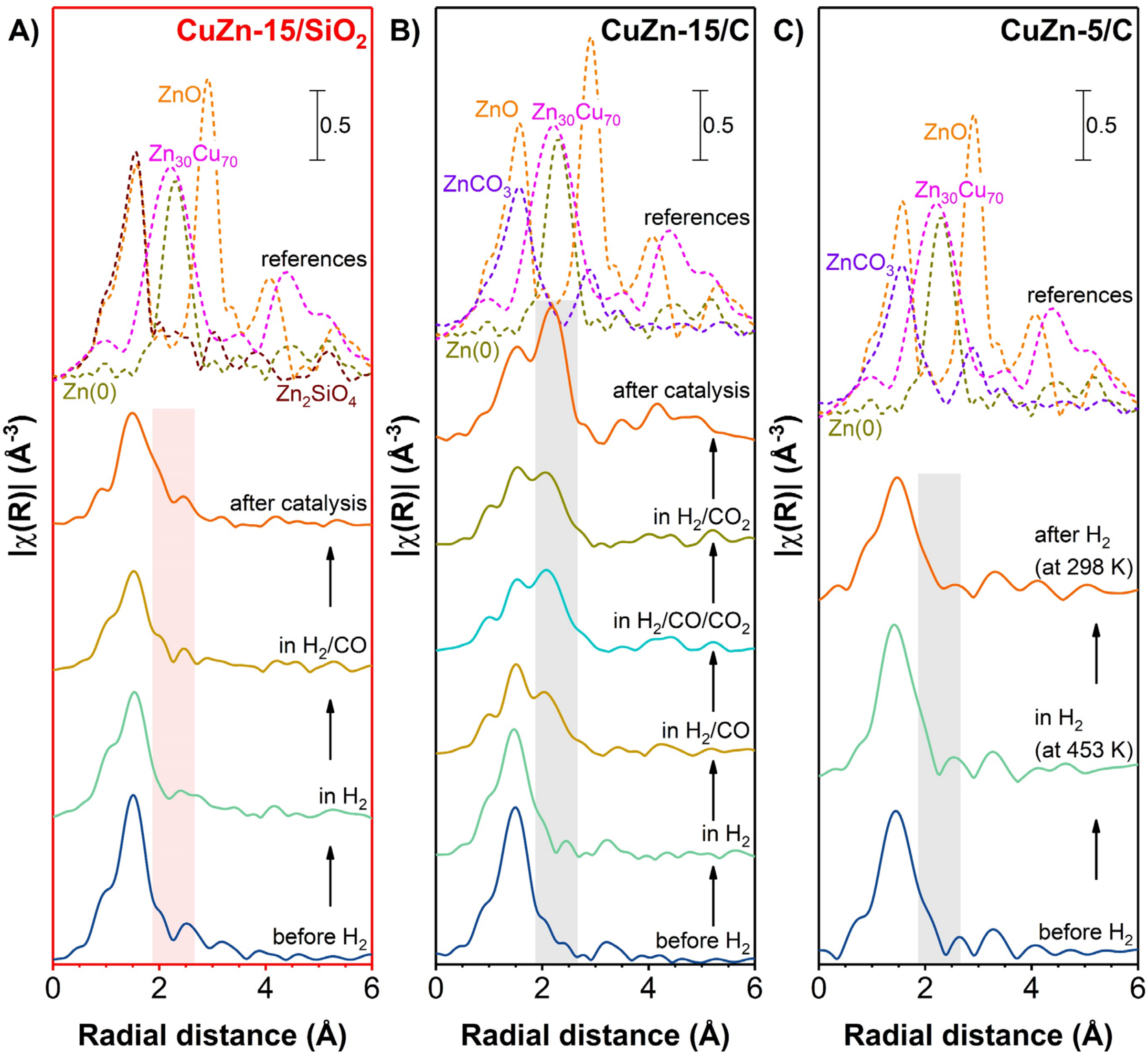

Figure S23 *Fourier-transformed EXAFS spectra at the Zn K-edge of the (**A**) CuZn-15/SiO*$_2$*, (**B**) CuZn-15/C and (**C**) CuZn-5/C catalysts (solid lines). Depicted (1) in the initial state at 298 K, (2) in an* H_2 *atmosphere at 453 K after an* H_2 *treatment (for conditions, see figure S16), (3-5) during a high-pressure* H_2/CO*,* $H_2/CO/CO_2$*, and* H_2/CO_2 *exposure at 533 K, respectively (for conditions, see figures S19-S20), and (6) after catalysis or* H_2 *treatment at 298 K. States (4) and (5) were only obtained for the CuZn-15/C catalyst. Dashed lines depict macrocrystalline ZnO,* Zn_2SiO_4*,* $ZnCO_3$*,* $Zn_{30}Cu_{70}$*, and Zn foil references at 298 K.*

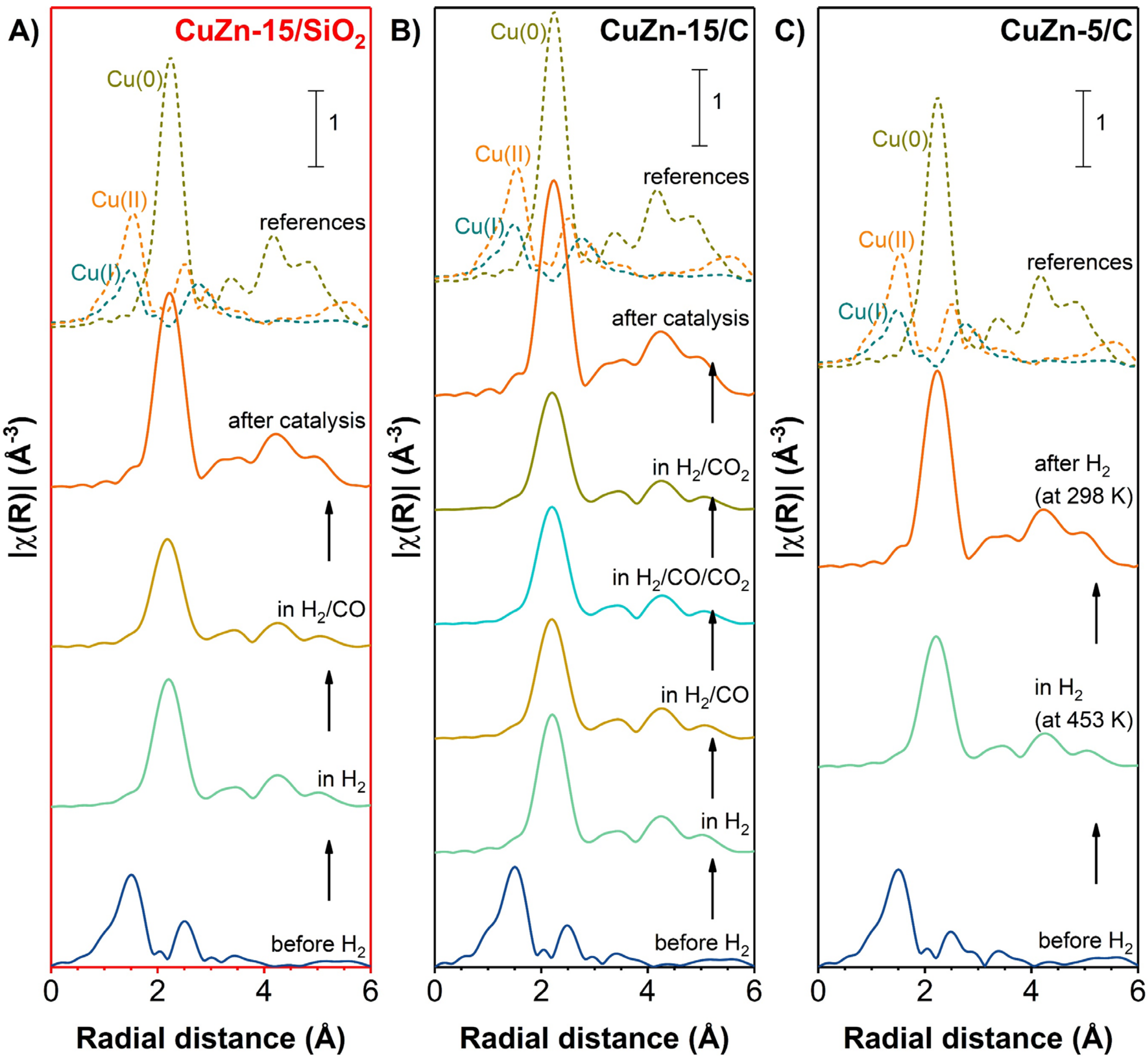

***Figure S24** Fourier-transformed EXAFS spectra at the Cu K-edge of the (**A**) CuZn-15/SiO_2, (**B**) CuZn-15/C and (**C**) CuZn-5/C catalysts (solid lines). Depicted (1) in the initial state at 298 K, (2) in an H_2 atmosphere at 453 K after an H_2 treatment (for conditions, see figure S16), (3-5) during a high-pressure H_2/CO, H_2/CO/CO_2, and H_2/CO_2 exposure at 533 K, respectively (for conditions, see figures S19-S20), and (6) after catalysis or H_2 treatment at 298 K. States (4) and (5) were only obtained for the CuZn-15/C catalyst. Dashed lines depict macrocrystalline CuO, Cu_2O, and Cu foil references at 298 K.*

Table S6 *EXAFS fitting results of the Cu- and Zn-based references at the corresponding K-edges.*

**reference**	***T* (K)**	**shell**	**CN**	S_0^2	**Δ*E*₀ (eV)**	***R* (Å)**	σ^2 **(Å²)**
CuO	298	Cu–O	4 (fixed)	0.86 ± 0.06	-1.5 ± 1.0	1.948	0.0042 ± 0.0009
Cu^0 foil	298	Cu–Cu	12 (fixed)	0.89 ± 0.05	4.4 ± 0.6	2.547	0.0089 ± 0.0005
ZnO	298	Zn–O	4 (fixed)	0.89 ± 0.05	4.2 ± 0.8	1.973	0.0044 ± 0.0008
Zn^0 foil	298	Zn–Zn	12 (fixed)	0.71 ± 0.07	2.7 ± 0.9	2.661	0.0132 ± 0.0009

Table S7 *EXAFS fitting results of the CuZn-15/SiO₂ catalyst at the Cu and Zn K-edges. Zn–M refers to indistinguishable Zn–Zn and Zn–Cu shells.*

**stage during XAS analysis**	***T* (K)**	**shell**	**CN**	ΔE_0 **(eV)**	***R* (Å)**	σ^2 **(Å²)**
before H_2	298	Cu–O	3.4 ± 0.3	-0.9 ± 1.1	1.940	0.0047 ± 0.0011
in H_2	453	Cu–Cu	10.8 ± 0.9	3.0 ± 0.8	2.529	0.0138 ± 0.0009
in H_2/CO	533	Cu–Cu	10.5 ± 0.8	2.7 ± 0.9	2.525	0.0153 ± 0.0011
after catalysis	298	Cu–Cu	10.5 ± 0.8	4.1 ± 0.7	2.539	0.0101 ± 0.0007

**stage during XAS analysis**	***T* (K)**	**shell**	**CN**	ΔE_0 **(eV)**	***R* (Å)**	σ^2 **(Å²)**
before H_2	298	Zn–O	4 ± 1	3.0 ± 1.0	1.969	0.0087 ± 0.0014
in H_2	453	Zn–O	3.7 ± 0.4	3.9 ± 1.4	1.967	0.0086 ± 0.0019
in H_2/CO	533	Zn–O	2.6 ± 0.5	2.4 ± 1.8	1.948	0.007 ± 0.002
		Zn–M	2.2 ± 1.4		2.55	0.008 ± 0.006
after catalysis	298	Zn–O	2.6 ± 0.5	2.6 ± 2.6	1.96	0.007 ± 0.003
		Zn–M	2.2 ± 1.4		2.55	0.011 ± 0.006

***Table S8** EXAFS fitting results of the CuZn-15/C catalyst at the Cu and Zn K-edges. Zn–M refers to indistinguishable Zn–Zn and Zn–Cu shells.*

**stage during XAS analysis**	***T* (K)**	**shell**	**CN**	**ΔE_0 (eV)**	***R* (Å)**	**σ^2 (Å^2)**
before H_2	298	Cu–O	3.7 ± 0.3	-0.8 ± 1.0	1.941	0.0043 ± 0.0009
in H_2	453	Cu–Cu	11.1 ± 1.0	3.2 ± 0.8	2.534	0.0134 ± 0.0010
in H_2/CO	533	Cu–Cu	11.3 ± 1.1	2.9 ± 0.9	2.534	0.0151 ± 0.0011
in H_2/CO/CO_2	533	Cu–Cu	11.3 ± 1.1	2.7 ± 0.9	2.535	0.0153 ± 0.0011
in H_2/CO_2	533	Cu–Cu	11.3 ± 0.8	2.9 ± 0.9	2.535	0.0152 ± 0.0011
after catalysis	298	Cu–Cu	11.3 ± 0.8	4.2 ± 0.7	2.547	0.0097 ± 0.0007

**stage during XAS analysis**	***T* (K)**	**shell**	**CN**	**ΔE_0 (eV)**	***R* (Å)**	**σ^2 (Å^2)**
before H_2	298	Zn–O	4 ± 1	0.8 ± 1.0	1.970	0.0088 ± 0.0013
in H_2	453	Zn–O	3.8 ± 0.5	1.0 ± 1.8	1.944	0.009 ± 0.002
in H_2/CO	533	Zn–O	1.8 ± 0.2	-1.5 ± 1.9	1.916	0.006 ± 0.002
		Zn–M	6.1 ± 1.3		2.544	0.020 ± 0.003
in H_2/CO/CO_2	533	Zn–O	1.7 ± 0.3	-1.8 ± 1.7	1.918	0.010 ± 0.004
		Zn–M	8.0 ± 1.4		2.544	0.020 ± 0.002
in H_2/CO_2	533	Zn–O	2.0 ± 0.2	-2.9 ± 1.8	1.916	0.010 ± 0.003
		Zn–M	8.0 ± 0.8		2.532	0.021 ± 0.002
after catalysis	298	Zn–O	2.0 ± 0.2	-1.5 ± 1.1	1.941	-0.036 ± 0.009
		Zn–M	8.0 ± 0.8		2.545	-0.010 ± 0.007

***Table S9** EXAFS fitting results of the CuZn-5/C catalyst at the Cu K-edge.*

**stage during XAS analysis**	***T* (K)**	**shell**	**CN**	**ΔE_0 (eV)**	***R* (Å)**	**σ^2 (Å^2)**
before H_2	298	Cu–O	3.68 ± 0.19	-1.2 ± 0.7	1.944	0.0046 ± 0.0006
in H_2	453	Cu–Cu	10.5 ± 0.8	3.3 ± 0.8	2.533	0.0138 ± 0.0010
after H_2	298	Cu–Cu	10.5 ± 0.8	4.2 ± 0.7	2.540	0.0100 ± 0.0007

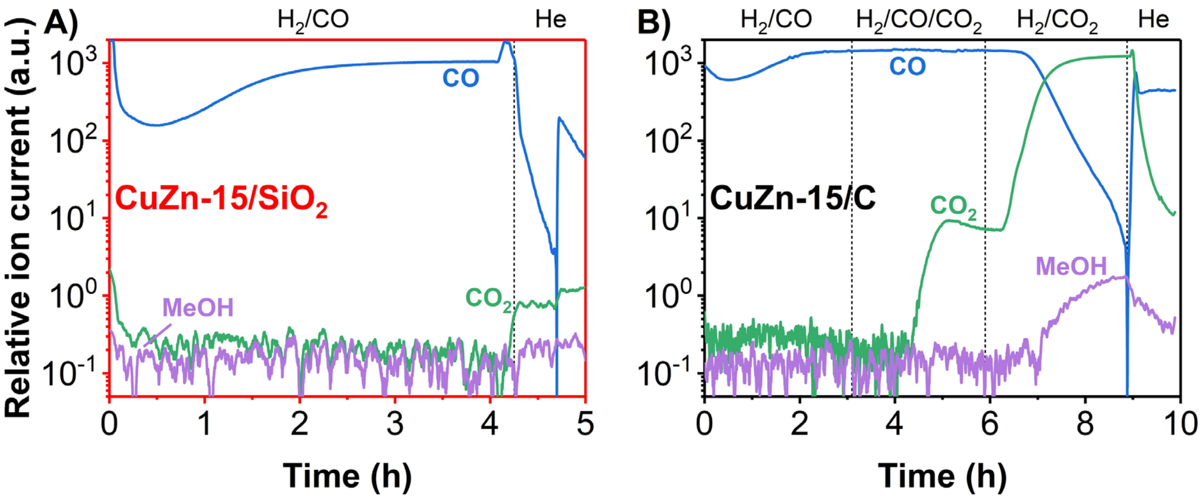

Figure S25 *MS data of the (**A**) CuZn-15/SiO_2 and (**B**) CuZn-15/C catalysts of the effluent of the capillary during syngas exposure at 20 bar, showing the changing gas compositions and methanol formation during catalysis. Note the delay up to 2 h between the change of gas feed and its detection by MS. Only a single carbonyl trap, which had been conditioned for the H_2/CO gas mixture, was used for both H_2/CO and H_2/CO/CO_2 gas mixtures. This explains the additional delay in the appearance of CO_2 in the H_2/CO/CO_2 feed compared to the appearance of CO_2 in the H_2/CO_2 feed. Both catalysts had a negligible activity in an H_2/CO feed.*

Identification of the exact composition and structure of the Zn spectator species in the CuZn-15/SiO_2 catalyst is challenging due to (1) the lack of long-range ordering evidenced by EXAFS analysis and XRD patterns, (2) the relatively low Zn loading of varying composition, and (3) the minor electronic variation between a Zn–O–Zn bond in ZnO and a Zn–O–Si bond in Zn_2SiO_4. Figure S26 shows additional infrared spectra using attenuated total reflection (ATR-IR) of selected catalysts and macrocrystalline references. No signal of supported Cu and Zn species was observed, as in line with the low metal loadings.

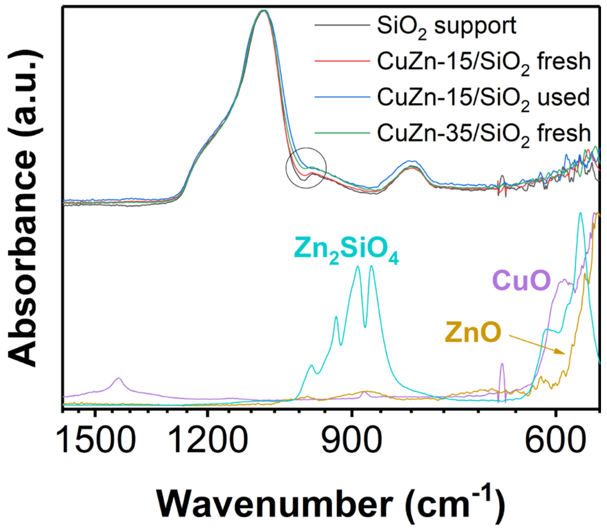

Figure S26 *ATR-IR spectra of selected $CuZnO_x/SiO_2$ catalysts, SiO_2 support, and macrocrystalline Zn_2SiO_4, ZnO, and CuO references. No Zn or Cu species were observed in the catalysts due to the low metal loadings and high SiO_2 amount. Conditions: apparatus = PerkinElmer Frontier FT-IR spectrophotometer, scan range = 4500-550 cm^{-1}, resolution = 2 cm^{-1}, scan averaging = 10 x 50 scans, $LiTaO_3$ detector with KBr windows.*

XAS fitting details. The XAS spectra were processed using the Demeter software package.[14] Spectra were normalized and background-subtracted by fitting a line to the pre-edge region and cubic splines to the post-edge region. Spectra were energy-aligned using the Cu and Zn metal foil reference spectra, located between the second and third ionization chambers, and measured simultaneously with the samples.

Prior to analyses of the XAS data at the XANES region, the number of principal components was assessed by principal component analysis (PCA) of the complete time series. In all cases the cumulative variance explained (CVE) of the first three principal components covered more than 99.99% of the variance of the data, suggesting that three principal components were sufficient to describe the data by multivariate curve resolution (MCR). Note that a principal component in PCA is not a pure chemical phase but can represent a linear combination of pure chemical components in a constant ratio throughout the time series.

The *in situ* XAS data at the XANES region was analyzed with linear combination fitting (LCF) using the Athena software. For the Cu K-edge, a spectrum of the macrocrystalline Cu_2O reference at 298 K was used for the Cu^+ state, while representative samples were used as references for the Cu^{2+} and Cu^0 states: a spectrum of the sample recorded at 298 K in a He atmosphere and recorded at 543 K in an H_2/He atmosphere, respectively. For the Zn K-edge, the average Zn oxidation number (ON) was

estimated using Zn metal foil as reference for the Zn^0 state and macrocrystalline ZnO and Zn_2SiO_4 as reference for the Zn^{2+} state. The LCF was performed in Athena on the normalized $\mu(E)$ spectra in the region between -20 to 30 eV from the absorption edge.

MCR with constraints was performed on the data matrix using alternating least squares (ALS), employing an in-house code written in Matlab®. MCR-ALS decomposes a matrix **X** such that **X** = **CS** + **E**, where **E** is minimized using ALS. Here, **X** represents the data arranged in a way that every row is a spectrum recorded at various times, *i.e.* different row index. MCR-ALS allows to set constraints for the matrix decomposition, which, in comparison to singular value decomposition (SVD) used in PCA, can help in obtaining more meaningful eigenspectra (or spectral profiles) in **S** in terms of spectra that more closely resemble spectra of pure chemical components. Here, the constraints that are especially useful in the analysis of time-resolved XAS data are non-negativity and closure, assuming that spectra do not contain negative values and that the concentration of the element studied is constant. MCR-ALS will then decompose the data matrix into non-negative eigenspectra in **S** and concentration profiles in **C** that sum to unity. We analyzed the data using these constraints and three components, as determined by PCA as mentioned before. Based on these results, we further varied the number of components between two and four and the number of iterations, thereby comparing the obtained MCR-ALS eigenspectra with the reference data, before selecting the final number of components and iterations.

The EXAFS data was fitted using the Artemis software. The amplitude reduction factor (S_0^2) was calculated from the EXAFS analysis of the known reference materials and used as a fixed parameter during the fitting procedure for the spectra recorded at room temperature. The coordination number (CN), energy correction term (ΔE_0), the Debye-Waller factor (σ^2), and the shift in the interatomic distance compared to the FEFF model structure (ΔR) were used as free parameters during the fitting. Multiple *k*-weight fittings were employed to derive the structural parameters. The typical distance fit range was 1.4-3.0 Å for Cu K-edge fitting and 1.0-2.3 Å for Zn K-edge fitting in the Fourier transform. No phase correction was applied.

Additional information during measurements. During the H_2 treatment (15 mL min^{-1} of 20 vol% H_2/He) the following temperature profiles were used with a heating rate of 5 K min^{-1}. For CuZn-15/SiO_2: from 298 K to 543 K, hold for 5 min. For CuZn-15/C: from 298 K to 523 K, hold for 20 min, then to 543 K, hold for 15 min. For CuZn-5/C: from 298 K to 543 K, hold for 16 min. XAS spectra

were recorded in each syngas atmosphere for 160 min but only for 120 min for the CuZn-15/C catalyst in the H_2/CO_2/He feed. The leak test for the CuZn-15/SiO_2 catalyst at 20 bar of He was performed after the *in situ* H_2 treatment.

Diffusion coefficients.

Table S10 *Calculated diffusion coefficients (in cm^2 s^{-1}) of Zn^0 into Cu^0 nanoparticles at different temperatures and Cu^0 particle sizes. The diffusion coefficient highly depends on the temperature and particle size: all Cu^0 particles smaller than 4.9 nm are fully alloyed with Zn^0 after 5 min of reduction at 543 K (as for the CuZn-15/SiO_2 catalyst). All Cu^0 particles smaller than 5.7 nm are fully alloyed after the slightly longer reduction at 543 K (as for the CuZn-15/C catalyst). See also references [15-17].*

Temperature (K)	3.8 nm Cu particles (as on CuZn-15/SiO_2)	4.6 nm Cu particles (as on CuZn-15/C)	10 nm Cu particles (as reference)
523	$2.1 \cdot 10^{-14}$	$1.7 \cdot 10^{-16}$	$2.6 \cdot 10^{-20}$
533	$3.7 \cdot 10^{-14}$	$3.3 \cdot 10^{-16}$	$5.9 \cdot 10^{-20}$
543	$6.5 \cdot 10^{-14}$	$6.2 \cdot 10^{-16}$	$1.3 \cdot 10^{-19}$

Zn_2SiO_4 and $ZnCO_3$ references. Zn_2SiO_4 was prepared *via* a solid-state reaction based on the review of Takesue *et al.*[18] ZnO (0.73 g, Puratronic®, abcr Chemicals, 99.999%) and an excess of silica gel (0.59 g, Davisil™, grade 643, Sigma Aldrich, ≥99%) (Zn/Si molar ratio of 1/1.1) were finely ground with a pestle and mortar for at least 20 min. The mixed powder was heated in a tubular furnace to 1373 K (ramp 5 K min^{-1}) in an N_2 flow for 4 h. After the solid-state reaction 1.25 g Zn_2SiO_4 was obtained and finely ground before use.

$ZnCO_3$ was prepared *via* a one-step hydrothermal method according to Pan *et al.*[19] $Zn(NO_3)_2 \cdot 6H_2O$ (3.00 g, Sigma Aldrich, ≥99%) and urea (0.70 g, Sigma Aldrich, ACS reagent, ≥99%) (molar ratio of 1/1.15) were dissolved in 75 mL MilliQ water and stirred vigorously at room temperature for 30 min. The solution was transferred to a 250 mL autoclave and heated in a muffle oven from room temperature to 413 K and held for 15 h. After slowly cooling down to room temperature the white precipitate was filtered by vacuum and washed with 1 L MilliQ and subsequently with 100 mL EtOH. After drying for several days in static air at 333 K, 0.98 g of $ZnCO_3$ was obtained (77% yield).

Figure S27 (frame A) shows the XRD patterns of the initial ZnO + SiO_2 mixture and the final Zn_2SiO_4 material. Almost only crystalline Zn_2SiO_4 was obtained with only a minor amount of the initial

powders present. The excess of silica gel probably led to a higher conversion level of the ZnO into Zn_2SiO_4, whereas the leftover amorphous SiO_2 did not influence the XAS data on the Zn K-edge. Frame B shows the XRD pattern of the obtained $ZnCO_3$ material. Both crystalline smithsonite ($ZnCO_3$) and hydrozincite ($Zn_5(CO_3)_2(OH)_6$) phases were present in the final powder, amongst probably other (amorphous) mixed zinc carbonate hydroxides and/or hydrates. Even though the synthesized material did not fully correspond to $ZnCO_3$, probably most compounds had the characteristic Zn–CO_3 bond, important for its characteristic features during XAS. All patterns were measured on a Bruker D8 Advance apparatus with Co Kα radiation (λ = 1.790 Å) at 30 kV and 40 mA with a variable slit intensity.

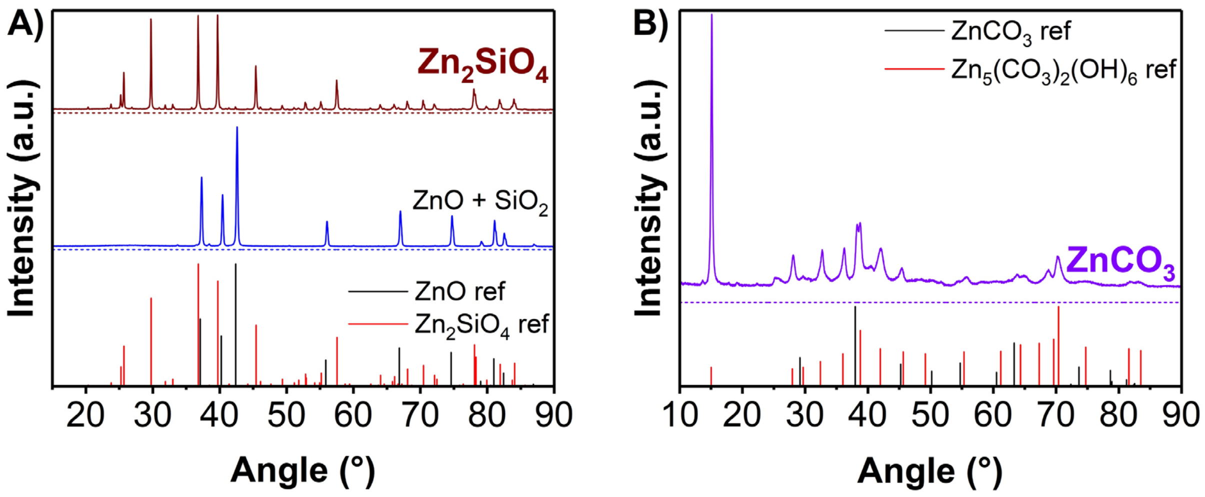

Figure S27 *(**A**) XRD patterns of the in-house synthesized Zn_2SiO_4 material and the initial ZnO + SiO_2 mixture (solid lines), alongside with ZnO and Zn_2SiO_4 reference data (bars). Note that the diffraction peaks of the amorphous silica gel (SiO_2) are barely visible. (**B**) XRD pattern of the prepared $ZnCO_3$ material (solid line) with $ZnCO_3$ (smithsonite) and $Zn_5(CO_3)_2(OH)_6$ (hydrozincite) reference data (bars).*

Section S5: Calculation of activity, selectivity, and stability

The individual CO and CO_2 conversions (X_{CO} and X_{CO2}) were calculated according to equation (S1), in which *A* represents the GC peak area of the corresponding compound obtained after the syngas mixture passed through a SiC-filled reference reactor (*i.e.* "in") or through a catalyst-filled reactor (*i.e.* "out"). The conversion levels were calibrated *via* He as an internal standard. The combined CO + CO_2 conversion (equation (S2)) was calculated by taking the volume fractions of CO (φ_{CO}) and CO_2 (φ_{CO2}) in the syngas into account. Alternatively, the conversion levels were also calculated from all products *i* formed (equation (S3)) as the carbon mass balance (C_{bal}) was *ca.* 100 ± 1.1%$_C$ during all

stages of catalysis (equation (S4)). Here, Y is the yield, Q the volumetric flow rate, RF the response factor, and N_C the number of carbon atoms.

$$X_{CO_{(2)}} = \frac{\left(A_{CO_{(2)}}/A_{He}\right)_{in} - \left(A_{CO_{(2)}}/A_{He}\right)_{out}}{\left(A_{CO_{(2)}}/A_{He}\right)_{in}} \cdot 100\% \ [\%] \quad (S1)$$

$$X_{CO+CO_2} = \frac{\varphi_{CO} \cdot X_{CO} + \varphi_{CO_2} \cdot X_{CO_2}}{\varphi_{CO} + \varphi_{CO_2}} \ [\%] \quad (S2)$$

$$X^{alt}_{CO\,(+\,CO_2)} = \sum_{i=1}^{N} Y_i = \frac{\left[\left(Q_{CO_2}\right)_{out} - \left(Q_{CO_2}\right)_{in}\right] + \sum_{i=1}^{N}(Q_i)_{out}}{(Q_{CO})_{in} + \left(Q_{CO_2}\right)_{in}} \cdot 100\% \ [\%] \quad (S3)$$

$$C_{bal} = 100\% - (C_{in} - C_{out}) = 100\% - \left(\frac{(Q_{CO})_{in} + \left(Q_{CO_2}\right)_{in}}{(Q_{tot})_{in}} \cdot 100\% - \frac{(A_{CO})_{out}}{RF_{CO}} + \frac{\left(A_{CO_2}\right)_{out}}{RF_{CO_2}} + \sum_{i=1}^{N} \frac{(A_i)_{out} \cdot N_{C,i}}{RF_i}\right) \ [\%] \quad (S4)$$

The Cu-normalized total activity and methanol formation rate were calculated *via* equations (S5)-(S6). Here, F_{CO} and F_{CO2} represent the molar flow rates in the reaction feed of CO and CO_2, respectively. The product selectivity S is calculated according to equation (S12). The stability of catalytic activity was calculated by equation (S7).

$$\text{Total activity} = \frac{X^{alt}_{CO\,(+\,CO_2)} \cdot \left(F_{CO} + F_{CO_2}\right)}{m_{Cu} \cdot 100\%} \ \left[\mu mol_{CO\,(+\,CO_2)\,conv.}\ g^{-1}_{Cu}\ s^{-1}\right] \quad (S5)$$

$$\text{Methanol formation rate} = \frac{\text{Total activity} \cdot S_{MeOH}}{100\%} \ \left[\mu mol_{MeOH\,form.}\ g^{-1}_{Cu}\ s^{-1}\right] \quad (S6)$$

$$\text{Stability} = \frac{X^{alt}_{CO\,(+\,CO_2)}(t = 100\ h)}{X^{alt}_{CO\,(+\,CO_2)}(t = 50\ h)} \cdot 100\% \ [\%] \quad (S7)$$

The turnover frequency (TOF) was determined at an initial stage (equation (S8)) and final stage (equation (S9)) of catalysis. The initial TOF was calculated from the surface-averaged Cu(Zn)O_x size in the fresh catalyst ($d_S^{initial}$) and the activity at t = 0 h, while the final TOF was obtained from the surface-

averaged Cu(Zn)O_x size after 100 or 150 h of catalysis (d_S^{final}) and the activity at the corresponding time. The methanol-specific TOF_{MeOH} was calculated according to equation (S10).

$$TOF_{initial} = \frac{X^{alt}_{CO\,(+\,CO_2)}(t = 0\text{ h}) \cdot \left(F_{CO} + F_{CO_2}\right)}{Cu_{surf}\left(d_S^{initial}, m_{Cu}\right)}\ \left[10^{-3}\ mol_{CO\,(+\,CO_2)\,conv.}\ mol^{-1}_{Cu_{surf}}\ s^{-1}\right] \quad (S8)$$

$$TOF_{final} = \frac{X^{alt}_{CO\,(+\,CO_2)}(t = 100\text{ h or }150\text{ h}) \cdot \left(F_{CO} + F_{CO_2}\right)}{Cu_{surf}\left(d_S^{final}, m_{Cu}\right)}\ \left[10^{-3}\ mol_{CO\,(+\,CO_2)\,conv.}\ mol^{-1}_{Cu_{surf}}\ s^{-1}\right] \quad (S9)$$

$$TOF_{MeOH} = \frac{TOF \cdot S_{MeOH}}{100\%}\ \left[10^{-3}\ mol_{MeOH\,form.}\ mol^{-1}_{Cu_{surf}}\ s^{-1}\right] \quad (S10)$$

The molar amount of Cu surface atoms in the catalyst (Cu_{surf}) used in equations (S8)-(S10) was calculated according to equation (S11), which depends on the Cu dispersion (D_{Cu}, the ratio between Cu surface atoms and the total amount of Cu atoms) and the molar amount of Cu atoms in the catalyst (n_{Cu}). In turn, the D_{Cu} depends on the Cu molar volume (V_m) and particle area (A_m), which are $7.09 \cdot 10^{21}$ nm^3 and $4.10 \cdot 10^{22}$ nm^2, respectively.[1]

$$Cu_{surf} = D_{Cu} \cdot n_{Cu} = \frac{6 \cdot V_m}{A_m \cdot d_S} \cdot n_{Cu} \approx \frac{1.04\text{ nm}}{d_S[\text{nm}]} \cdot n_{Cu}\ [mol_{Cu_{surf}}] \quad (S11)$$

The carbon atom-based methanol selectivity (S_{MeOH}) was calculated according to equation (S12). CO_2 was excluded as a possible product in the product distribution.

$$S_{MeOH} = \frac{A_{MeOH}}{RF_{MeOH}}\left[\sum_{i=1}^{N} \frac{A_i \cdot N_{C,i}}{RF_i}\right]^{-1} \cdot 100\%\ [\%_C] \quad (S12)$$

References

(1) Dalebout, R.; Visser, N. L.; Pompe, C. E.; de Jong, K. P.; de Jongh, P. E. Interplay between Carbon Dioxide Enrichment and Zinc Oxide Promotion of Copper Catalysts in Methanol Synthesis. *J. Catal.* **2020**, *392*, 150–158.

(2) Robinson, W. R. A. M.; Mol, J. C. Support Effects in Methanol Synthesis over Copper-Containing Catalysts. *Appl. Catal.* **1991**, *76* (1), 117–129.

(3) van den Berg, R.; Prieto, G.; Korpershoek, G.; van der Wal, L. I.; van Bunningen, A. J.; Lægsgaard-Jørgensen, S.; de Jongh, P. E.; de Jong, K. P. Structure Sensitivity of Cu and CuZn Catalysts Relevant to Industrial Methanol Synthesis. *Nat. Commun.* **2016**, *7*, 13057.

(4) Prieto, G.; Zečević, J.; Friedrich, H.; de Jong, K. P.; de Jongh, P. E. Towards Stable Catalysts by Controlling Collective Properties of Supported Metal Nanoparticles. *Nat. Mater.* **2013**, *12* (1), 34–39.

(5) van den Bleek, C. M.; van der Wiele, K.; van den Berg, P. J. The Effect of Dilution on the Degree of Conversion in Fixed Bed Catalytic Reactors. *Chem. Eng. Sci.* **1969**, *24* (4), 681–694.

(6) Kurtz, M.; Strunk, J.; Hinrichsen, O.; Muhler, M.; Fink, K.; Meyer, B.; Wöll, C. Active Sites on Oxide Surfaces: ZnO-Catalyzed Synthesis of Methanol from CO and H_2. *Angew. Chem. Int. Ed.* **2005**, *44* (18), 2790–2794.

(7) Pompe, C.E.; van Uunen, D.L.; van der Wal, L.I.; van der Hoeven, J.E.S.; de Jong, K.P.; de Jongh, P.E. Stability of Mesocellular Foam Supported Copper Catalysts for Methanol Synthesis. *Catal. Today* **2019**, *334*, 79–89.

(8) Fichtl, M.B.; Schlereth, D.; Jacobsen, N.; Kasatkin, I.; Schumann, J.; Behrens, M.; Schlögl, R. Kinetics of Deactivation on Cu/ZnO/Al_2O_3 Methanol Synthesis Catalysts. *Appl. Catal. A* **2015**, *502*, 262–270.

(9) Lunkenbein, T.; Girgsdies, F.; Kandemir, T.; Thomas, N.; Behrens, M.; Schlögl, R.; Frei, E. Bridging the Time Gap: A Copper/Zinc Oxide/Aluminum Oxide Catalyst for Methanol Synthesis Studied under Industrially Relevant Conditions and Time Scales. *Angew. Chem. Int. Ed.* **2016**, *55* (41), 12708–12712.

(10) Li, C.; Yuan, X.; Fujimoto, K. Development of Highly Stable Catalyst for Methanol Synthesis from Carbon Dioxide. *Appl. Catal. A* **2014**, *469*, 306–311.

(11) Prašnikar, A.; Pavlišič, A.; Ruiz Zepeda, F.; Kovač, J.; Likozar, B. Mechanisms of Copper-Based Catalyst Deactivation during CO_2 Reduction to Methanol. *Ind. Eng. Chem. Res.* **2019**, *58*, 13021–13029.

(12) Kühl,S.; Tarasov, A.; Zander, S.; Kasatkin, I.; Behrens, M. Cu-Based Catalyst Resulting from a Cu,Zn,Al Hydrotalcite-Like Compound: A Microstructural, Thermoanalytical, and In Situ XAS Study. *Chem. Eur. J.* **2014**, *20* (13), 3782–3792.

(13) Schütte K.; Meyer, H.; Gemel, C.; Barthel, J.; Fischer, R.A.; Janiak, C. Synthesis of Cu, Zn and Cu/Zn Brass Alloy Nanoparticles from Metal Amidinate Precursors in Ionic Liquids or Propylene Carbonate with Relevance to Methanol Synthesis. *Nanoscale* **2014**, *6* (6), 3116–3126.

(14) Ravel, B.; Newville, M. ATHENA, ARTEMIS, HEPHAESTUS: Data Analysis for X-ray Absorption Spectroscopy using IFEFFIT. *J. Synchrotron Rad.* **2005**, *12* (4), 537–541.

(15) Spencer, M. S. α-Brass Formation in Copper/Zinc Oxide Catalysts: II. Diffusion of Zinc in Copper and α-Brass under Reaction Conditions. *Surf. Sci.* **1987**, *192* (2–3), 329–335.

(16) Guisbiers, G.; Buchaillot, L. Size and Shape Effects on Creep and Diffusion at the Nanoscale. *Nanotechnology* **2008**, *19* (43), 435701.

(17) Guisbiers, G.; Kazan, M.; van Overschelde, O.; Wautelet, M.; Pereira, S. Mechanical and Thermal Properties of Metallic and Semiconductive Nanostructures. *J. Phys. Chem. C* **2008**, *112* (11), 4097–4103.

(18) Takesue, M.; Hayashi, H.; Smith Jr., R.L. Thermal and Chemical Methods for Producing Zinc Silicate (Willemite): A Review. *Prog. Cryst. Growth Charact. Mater.* **2009**, *55* (3–4), 98–124.

(19) Pan, Y.-T.; Wang, D.-Y. One-Step Hydrothermal Synthesis of Nano Zinc Carbonate and Its Use as a Promising Substitute for Antimony Trioxide in Flame Retardant Flexible Poly(Vinyl Chloride). *RSC Adv.* **2015**, *5* (35), 27837–27843.
